# Cluster-specific associations between the gut microbiota and behavioral outcomes in preschool-aged children

**DOI:** 10.1186/s40168-024-01773-5

**Published:** 2024-03-21

**Authors:** Marcel van de Wouw, Yanan Wang, Matthew L. Workentine, Elnaz Vaghef-Mehrabani, Delaney Barth, Emily M. Mercer, Deborah Dewey, Marie-Claire Arrieta, Raylene A. Reimer, Lianne Tomfohr-Madsen, Gerald F. Giesbrecht

**Affiliations:** 1 Department of Pediatrics, University of Calgary, Calgary, Alberta Canada; 2 Department of Psychology, University of Calgary, Calgary, Alberta Canada; 3 Alberta Children’s Hospital Research Institute (ACHRI), University of Calgary, Calgary, Alberta Canada; 4 Department of Community Health Sciences, University of Calgary, Calgary, Alberta Canada; 5 Faculty of Veterinary Medicine, UCVM Bioinformatics, University of Calgary, Calgary, Alberta Canada; 6 Faculty of Kinesiology, University of Calgary, Calgary, Alberta Canada; 7 Department of Biochemistry and Molecular Biology, Cumming School of Medicine, University of Calgary, Alberta, Canada; 8 Microbiomes for One Systems Health, Health & Biosecurity, CSIRO, Adelaide, SA Australia; 9 Department of Physiology and Pharmacology, University of Calgary, Calgary, AB Canada; 10 International Microbiome Centre, University of Calgary, Calgary, Alberta Canada; 11 Hotchkiss Brain Institute (HBI), University of Calgary, Calgary, Alberta Canada; 12 Faculty of Education, University of British Columbia, Vancouver, British Columbia Canada

**Keywords:** Gut microbiota, Cluster, Child, Neurodevelopment, Gut-brain axis, Alberta Pregnancy Outcomes and Nutrition (APrON) Study, Sociability, Internalizing, Externalizing

## Abstract

**Background:**

The gut microbiota is recognized as a regulator of brain development and behavioral outcomes during childhood. Nonetheless, associations between the gut microbiota and behavior are often inconsistent among studies in humans, perhaps because many host-microbe relationships vary widely between individuals. This study aims to stratify children based on their gut microbiota composition (i.e., clusters) and to identify novel gut microbiome cluster-specific associations between the stool metabolomic pathways and child behavioral outcomes.

**Methods:**

Stool samples were collected from a community sample of 248 typically developing children (3–5 years). The gut microbiota was analyzed using 16S sequencing while LC-MS/MS was used for untargeted metabolomics. Parent-reported behavioral outcomes (i.e., Adaptive Skills, Internalizing, Externalizing, Behavioral Symptoms, Developmental Social Disorders) were assessed using the Behavior Assessment System for Children (BASC-2). Children were grouped based on their gut microbiota composition using the Dirichlet multinomial method, after which differences in the metabolome and behavioral outcomes were investigated.

**Results:**

Four different gut microbiota clusters were identified, where the cluster enriched in both *Bacteroides* and *Bifidobacterium* (Ba2) had the most distinct stool metabolome. The cluster characterized by high *Bifidobacterium* abundance (Bif), as well as cluster Ba2, were associated with lower Adaptive Skill scores and its subcomponent Social Skills. Cluster Ba2 also had significantly lower stool histidine to urocanate turnover, which in turn was associated with lower Social Skill scores in a cluster-dependent manner. Finally, cluster Ba2 had increased levels of compounds involved in Galactose metabolism (i.e., stachyose, raffinose, alpha-D-glucose), where alpha-D-glucose was associated with the Adaptive Skill subcomponent Daily Living scores (i.e., ability to perform basic everyday tasks) in a cluster-dependent manner.

**Conclusions:**

These data show novel associations between the gut microbiota, its metabolites, and behavioral outcomes in typically developing preschool-aged children. Our results support the concept that cluster-based groupings could be used to develop more personalized interventions to support child behavioral outcomes.

Video Abstract

**Supplementary Information:**

The online version contains supplementary material available at 10.1186/s40168-024-01773-5.

## Background

There is growing recognition that the gut microbiota during early life can influence neurodevelopmental and behavioral outcomes [1, 2]. Many studies have reported links between the gut microbiota and behavioral outcomes in children; however, many of these associations are inconsistent among studies. For instance, one study reported a negative association between alpha diversity and internalizing problem scores (i.e., behaviors directed inward such as anxiety or depression) on the Child Behavior Checklist (CBCL) in preschool-aged children [3], while another study reported a positive association between alpha diversity and behavioral problems measured using the CBCL during the first 2 years of life [4]. A more recent article also reported differential sex-dependent associations between alpha diversity at 2 years of age with Adaptive Skill scores (i.e., emotional expression and control, daily living skills, and communication skills) measured using the Behavioral Assessment System for Children (BASC-2) at 3 years of age [5].

In tandem, specific gut microbial taxa have been linked to behavior, social skills, and cognition [6]. For instance, *Bifidobacterium* sp. SV1, *Bacteroides vulgatus* SV11, and *Streptococcus* sp. SV217 is positively associated with Adaptive Skill scores measured using the BASC-2 at 3 years of age in boys, but not girls, whereas *Klebsiella* sp. SV20, *Clostridium* sp. SV41, and *Haemophilus* sp. SV415 are negatively associated [5]. In addition, reduced fecal *Prevotella* abundance at 12 months of age is associated with internalizing problems at 2 years of age [4]. There is a negative association between *B. fragilis* and *B*. *thetaiotaomicron* relative abundance with externalizing behaviours (i.e., behaviors directed outwards such as aggression or hyperactivity) measured using the CBCL in children at 5 to 7 years of age [7], whereas *Prevotella* abundance during childhood was positively associated with externalizing behaviors measured using the CBCL at 6 years of age [8]. Overall, various associations have been identified between the composition of the gut microbiota and behavioral outcomes throughout childhood, although discrepancies across studies remain. Some of the noted inconsistencies at the gut microbial composition level could be explained by the fact that microbial processes can be performed by multiple different bacteria (i.e., functional redundancy) [9]. Metabolomics analysis could be used to enhance the understanding of the link between microbial processes and behavioral outcomes. Discrepancies between studies could further result from individual variability in how the gut microbiome signals to the brain, and stratifying individuals based on their gut microbiota or diet may provide insights into such individualized relationships, and perhaps also more effective microbiota-targeted interventions across several disorders [10–13]. Overall, it is important that we understand the associations between the gut microbiome and child behavior, as child behavior is strongly predictive of mental health status in later life [14, 15], and identifying (gut microbiome) interventions during early life that support child development may therefore improve mental health outcomes in later life.

One method that has expanded our understanding of the role of gut microbiota in health and disease is to stratify individuals into groups based on the composition of their gut microbiota, also often called clusters [16, 17]. In adults, this typically results in three to four distinct clusters, which are often characterized by higher relative abundances of the bacterial taxa *Prevotella*, *Bacillota* (previously *Firmicutes*), and *Bacteroides*, where the latter cluster is often split into clusters Ba1 and Ba2 [17]. These clusters have already been linked to specific health outcomes in adults, such as cluster Ba2 being associated with increased systemic inflammation, lower cardiac vagal function, reduced quality of life scores, and increased odds for obesity, type 2 diabetes, Crohn’s disease and depression [18–23]. In children, a cluster enriched in *Bifidobacterium* and *Enterobacteriaceae* at 2.5 months of age was associated with elevated scores in the temperament trait of regulation using the Infant Behavior Questionnaire-Revised (IBQ-R) at 6 months of age [24]. Interestingly, one article reported that the relative abundance of *Bacteroides* spp. negatively correlates with plasma branched-chain amino acids in the cluster enriched in *Bacteroides* specifically [25], suggesting that some relationships between the gut microbiome and host physiology might be cluster-dependent. This is supported by the finding that associations between the gut microbiome and diet, or even drug metabolism, vary widely between individuals [26, 27]. Therefore, cluster-based groupings may allow us to identify associations between the gut microbiome and host physiology/behavior that would be difficult to identify without stratifying a study population. Understanding individualized microbiota-gut-brain axis associations may allow for tailored microbiota-targeted interventions for individuals, which may in turn enhance treatment efficacy across a number of disorders.

As such, the objective of this study was to identify novel cluster-dependent associations between the gut microbiome and its metabolites (i.e., stool metabolome) with behavioral outcomes in preschool children. Such findings could provide novel insights into microbiota-based personalized interventions for supporting behavior and mental health outcomes [13].

## Methods

### Participant recruitment

Participants were drawn from the Alberta Pregnancy Outcomes and Nutrition (APrON) study [28, 29], an ongoing longitudinal cohort study that initially recruited pregnant individuals < 27 weeks gestation. Pregnant individuals were excluded if they could not read or speak English, were less than 16 years of age, or were planning to move out of the recruitment region prior to birth (which would prevent follow-up). At 3–4 years of child age, parents and children were invited to participate in a neurocognitive and behavioral assessment and collect a stool sample. Children were excluded if they were diagnosed with neurocognitive deficits (including suspected diagnosis of autism) or if they had recent antibiotic exposure (2 weeks before the fecal sample collection). Children were asked to provide assent and a parent or legal guardian provided informed written consent before sample and data collection. Ethics approval for this study was obtained from the Health Research Ethics Boards at the University of Calgary (E22101).

### Assessment of child behavior

Child behavior was assessed using the parent-reported Behavior Assessment System for Children (BASC-2) [30]. The BASC-2 includes behavior scales for Adaptive Skills, Internalizing, Externalizing, Behavioural Symptoms, and Developmental Social Disorders (Table 1). The BASC-2 is reliable and valid, with an item consistency in general norm samples with Cronbach's alpha ranging from 0.87 to 0.93 for the primary behavioral scales [30]. T scores were used in the analyses. The Adaptive Skills scale includes the subcomponents, Social Skills, Functional Communication, Daily Living, and Adaptability.

**Table 1:** Interpretation of the behavioral scales

Behavioral scale	Subcomponent	Directionality	Interpretation
Internalizing	No	Higher = more problems	This composite reflects behaviors that are acted inwards (i.e., anxiety, depression, somatization)
Externalizing	No	Higher = more problems	This composite reflects behaviors that are acted outwards (i.e., hyperactivity, aggression)
Behavioral symptoms	No	Higher = more problems	This composite reflects the overall level of problem behavior (i.e., hyperactivity, aggression, depression, attention problems, atypicality, withdrawal).
Developmental social disorder	No	Higher = more problems	Tendency to display behaviors characterized by deficits in social skills, communication, interests, and activities. Sometimes used to rate autism-related behaviors.
Adaptive skills	No	Higher = more adaptive	This composite summarizes appropriate emotional expression and control, daily living skills, and communication skills.
Social skills	Yes, to adaptive skills	Higher = more adaptive	The skills necessary for interacting successfully with peers and adults in home, school, and community settings.
Functional communication	Yes, to adaptive skills	Higher = more adaptive	The ability to express ideas and communicate in a way others can easily understand.
Daily living	Yes, to adaptive skills	Higher = more adaptive	Skills associated with performing basic, everyday tasks in an acceptable and safe manner.
Adaptability	Yes, to adaptive skills	Higher = more adaptive	The ability to adapt readily to changes in the environment.

### Stool sample collection and 16S rRNA amplicon sequencing

Child stool samples were collected by parents at home at 3–5 years of age using a provided toilet cover along with a sterile 50-ml plastic conical collection tube and plastic applicator. Collected fecal samples were temporarily stored in a home freezer (− 20 °C) for up to 24 h before transport to the study lab in a cooler surrounded by freezer packs. Fecal samples were stored at – 80 ^◦^C until further processing.

Fecal DNA was extracted using FastDNA^®^ Spin Kit for Feces (MP Biomedicals, Santa Ana, CA, USA) following the manufacturer’s instructions. 16S rRNA gene amplicon sequencing was performed using the MiSeq platform at the Centre for Health Genomics and Informatics (University of Calgary, Calgary, Canada) as previously described [31]. The length of the amplicons was ~ 460 bp. The PCR procedure was as follows: (1) 95 °C for 3 min, (2) 25 cycles of 95 °C for 30 s, 55°C for 30 s, 72 °C for 30 s, (3) 72 °C for 5 min, (4) hold at 4 °C. The PCR amplification of the V3 and V4 region of the 16S rRNA gene was performed using manufacturer-recommended primers (Forward: 5′-TCGTCGGCAGCGTCAGATGTGTATAAGAGACAGCCTACGGGNGGCWGCAG-3′; Reverse: 5′-GTCTCGTGGGCTCGGAGATGTGTATAAGAGACAGGACTACHVGGGT ATCTAATCC-3′).

After a quality check with FastQC 0.11.5 and MultiQC 1.0 primers [32], low-quality sequences were trimmed off the raw sequence reads using cutadapt 1.14 [33]. The trimmed reads were used to construct amplicon sequence variants (ASVs) using dada2 1.10.0 in R 3.5.1 [34, 35]. Unless otherwise stated all dada2 functions were used with default parameters. Reads were first filtered with dada2::filterAndTrim with a max expected error of 1. Error rates were learned for the forward and reverse reads separately and these error rates were used to infer exact sequences (error correct) for each sample from dereplicated, trimmed reads using pooled=TRUE for the dada2::dada. Following this, the forward and reverse reads were merged using dada2::mergePairs. Chimeras were removed with dada2::removeBimeraDenovo and taxonomy was assigned using the naïve Bayesian classifier [36], as implemented in dada2::assignTaxonomy trained with the Silva training set version 132 [37]. Species-level assignment was done with dada2::addSpecies which uses exact matching to assign species where possible. ASVs were aligned with ssu-align 0.1.1 and a phylogenetic tree was constructed with FastTree 2.1.9 [38, 39]. Sequences matching mitochondria or chloroplast were removed along with any sequences that weren’t assigned to Bacteria. A filtered copy of the ASV sequence table was created that retained ASVs present (count >= 2) in at least 1% of the samples. This served to reduce noise for downstream analysis.

To calculate beta diversity, the ASV counts (filtered table) were normalized with a variance stabilizing transform (using DESeq2 1.24.0) with size factors calculated using GMPR. Then sample-sample distances were determined with the Bray-Curtis metric and visualized with detrended correspondence analysis (DCA) [40, 41]. Alpha diversity (observed OTUs, phylogenetic diversity, Shannon diversity) and gut bacterial taxa, of which 74 were detected that were present in more than 5% of all samples, were used in the analysis.

Gut microbiota clustering was done using Dirichlet multinomial mixtures [42]. Clustering was performed using count data at the genus level, with no set abundance/prevalence threshold (i.e., rare taxa were included). The optimal number of clusters was determined using Laplace approximation [42], which suggested a 4-cluster approach (Fig. S1A–D). Even though the Bayesian Information Criterion (BIC) and Aikaike Information Criterion (AIC) suggested that a 2-cluster approach may be more optimal, the Laplace approximation was chosen because the AIC and BIC can give misleading results [42, 43]. Comparison of the 2- and 4-cluster approaches showed considerable overlap in participant allocation between the approaches (Fig. S1E), suggesting that the 4-cluster approach encompasses the 2-cluster approach while being more nuanced. Gut microbial beta diversity between clusters was investigated using a permutational analysis of variance (PERMANOVA).

### LC-MS/MS-based metabolite quantification in fecal samples

Metabolomics analyses were performed at the Calgary Metabolomics Research Facility. For targeted short-chain fatty acids analyses (i.e., acetate, propionate, butyrate, isobutyrate, valerate), samples were processed as previously discussed [44, 45], except for the following modifications related to the additional quantification of native isobutyric and valeric acids. In brief, SCFAs were extracted (1:2 ratio wet sample weight (mg) to extraction solvent (μL)) from fecal samples with ice-cold extraction solvent (50% water/acetonitrile, v/v) spiked with stable isotope-labeled internal standards (IS) (acetic acid-1,2-13C2, 4 mM, final concentration; propionic acid-13C3, 1 mM; butyric acid-1,2-13C2, 1 mM; isobutyric acid-d7, 250 μM and valeric acid-d9, 500 μM), homogenized at 30 Hz for 3 min with a tissue lyser (Qiagen), derivatized with N-(3-Dimethylaminopropyl)-N′-ethylcarbodiimide hydrochloride (EDC) and aniline, then submitted to LC-MS/MS analysis. The UHPLC-MS platform consisted of a VanquishTM ultra-high-performance liquid chromatography system coupled to a TSQ QuantumTM Access MAX triple quadrupole Mass Spectrometer (Thermo Scientific) equipped with an electrospray ionization (HESI-II) probe. In short, derivatized SCFAs were separated on a Hypersil GOLD TM C18 column (200 × 2.1 mm, 1.9 μm, Thermo Scientific) using a binary solvent system composed of LC-MS grade water (A) and methanol (B) both containing 0.1% (%v/v) formic acid. The following modified 21 min gradient was used: 0–1 min, 10%B; 1–1.1 min, 40%B; 1.1–11 min, 40–98%B; 11–16 min, 98%B; 16–16.5 min, 98–10%B; 16.5–21 min, 10%B. LC eluent was diverted to waste for the first 5 min of the run. The derivatized SCFAs were monitored with the mass spectrometer operating in positive ionization mode and selected reaction monitoring (SRM) mode. The following transitions, corresponding to the five derivatized native SCFAs and respective derivatized 13C- or deuterated standards, were monitored, with a scan time of 0.05 sec and a fixed collision energy of 14 eV (12C- or 13C-analytes): [M+H]+ m/z 136.07, 138.08, 150.09, 153.10, 164.10, 166.11, 178.12 → m/z 94.06, or 18eV (deuterated analytes): [M+H]+ m/z 171.15, 187.18 → m/z 95.14. Data analyses, on the converted mzXML files, were conducted in MAVEN [46, 47], and the absolute quantification of native SCFA concentration was based on the 12C:IS signal intensity ratio and the respective internal standard concentration.

For untargeted metabolomics analyses, fecal samples (100–200 mg) were diluted 5 times (w/v) into 50% methanol/water solution. First, diluted samples were homogenized using Tissue Lyser II (QIAGEN) and were then incubated on ice for 30 min for full extraction. Next, samples were centrifuged (approximately 13,000 rpm) and 500 µl of supernatant was collected for metabolomic analysis. Q Exactive™ HF Hybrid Quadrupole-Orbitrap™ Mass Spectrometer (Thermo-Fisher) and Vanquish™ UHPLC System (Thermo-Fisher) were used to perform metabolomics runs. Chromatographic separation was done on a Syncronis HILIC UHPLC column (2.1 mm × 100 mm × 1.7 μm, Thermo-Fisher) using a binary solvent system at a flow rate of 600 μL/min. Analysis of metabolite data was done with El-MAVEN software package. Identification of metabolites was done by matching observed m/z signals (± 10 ppm) and chromatographic retention times to those observed from commercial metabolite standards (Sigma-Aldrich). An automated feature detection function in EL-MAVEN with a minimum signal intensity threshold of 50,000 signal intensity was used to generate raw untargeted data. Metabolite data was CLR-transformed.

### Dietary assessment

Parents completed a 100-item semi-quantitative Food Frequency Questionnaire (FFQ) for their child at 3 years of age. The FFQ lists food items that are commonly eaten by preschool-aged children, and the parents indicated how often their children consumed each of the items. The parents also reported on foods that their children consumed that were not on the FFQ list [48]. The FFQ was used to generate a previously validated diet quality index score (DQI) where higher values indicate greater adherence to the recommended values from Canada’s Food Guide 2007 [49]. As the diets of children are relatively stable at the ages of 3–4 [50], the dietary assessments conducted at 3 years of age in this study can be considered as a proxy for dietary intakes at 4 years of age, the timepoint at which the gut microbiome of the study participants was assessed.

### Statistical analysis

Gut microbiota cluster-dependent associations between the stool metabolome and behavioral outcomes were investigated as follows (see Fig. S2 for flowchart). Cluster and sample characteristics were described and sample characteristics that differed between clusters were included as covariates in follow-up analyses. Differences in individual stool metabolites and metabolic pathways (identified using MetaboAnalyst) were subsequently investigated, as well as behavioral differences between clusters. Finally, gut microbiota-dependent associations between the stool metabolome and behavioral outcomes were investigated.

Differences in participant characteristics between clusters were assessed using a one-way analysis of variance (ANOVA). Differences in stool metabolites and behavior between clusters were assessed using Kruskal-Wallis tests followed by Mann-Whitney tests. Differences in stool metabolome beta diversity between clusters were assessed using the Atchinson distance [51], where metabolite data was centered log-ratio transformed. Associations between gut microbial taxa, metabolites, and behavior were assessed using non-parametric Spearman correlations. MetaboAnalyst was used for metabolic pathway analyses, which groups individual metabolites into pathways and assesses whether metabolic pathways are associated with outcomes [52]. Controlling for covariates in the behavioral outcome comparisons between clusters was performed using an analysis of covariance (ANCOVA). Because of the paucity of data guiding the selection of covariates that may be important to gut microbiota clusters at preschool age, we used a data-driven approach for covariate selection by investigating which covariates differ between gut microbiota clusters using an ANOVA (*p* < 0.05). The following covariates differed between gut microbiota clusters and were included in the models: grain intake, ethnicity, gestational age at birth, and birth weight. Controlling for covariates in the cluster-specific associations between stool metabolites and behavioural outcomes was performed using partial correlation analyses. Pathways of interest were also determined by manually identifying metabolites that are directly metabolized into each other using the Kyoto Encyclopedia of Genes and Genomes (KEGG) database [53]. *P* values subsequently underwent a false-discovery rate (FDR) correction using the Benjamini and Hochberg method [54], where a *q* < 0.05 was deemed significant. SPSS software version 26 (IBM Corp) was used for statistical analyses.

## Results

### Cluster characteristics

**Fig. 1:**
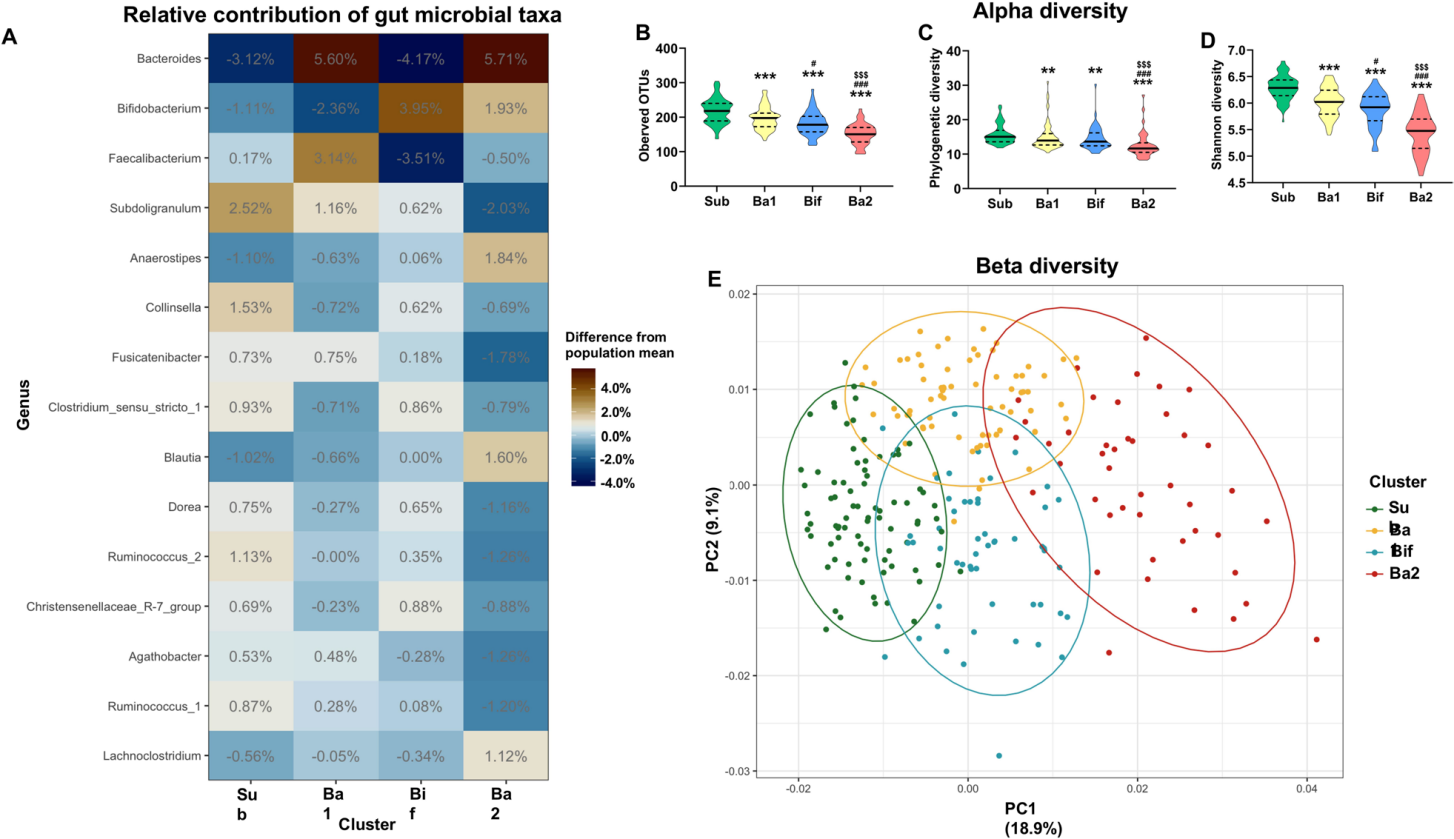
Gut microbiota composition differences between the four clusters. **A** Relative abundance differences between gut microbial taxa and their contributions to the cluster groupings were calculated. **B**–**D** Alpha diversity measures were compared between the clusters, and differences were assessed using Kruskal-Wallis tests followed by Mann-Whitney tests. Data are depicted as violin plots where the middle line is the median and the dotted lines are quartiles. **E** Differences in beta diversity were assessed, where individual dots are datapoints. Total *n* = 248, cluster sub is *n* = 81, Ba1 is *n* = 70, Bif is *n* = 53, Ba2 is *n* = 44. For the statistical significance, *p < 0.05; **p < 0.01; ***p < 0.001, * indicates a statistical difference compared to the sub cluster, # compared to the Ba1 cluster, $ compared to the Bif cluster

Assessment of cluster distribution using Dirichlet multinomial mixtures revealed 4 clusters (Fig. S1) [42]. The 15 most important taxa contributing to the differentiation of the clusters are displayed in order of their importance in Fig. 1A. *Bacteroides*, *Bifidobacterium*, and *Faecalibacterium* made the largest contributions to cluster separation. Cluster Sub was characterized by a high relative abundance of the genus *Subdoligranulum*. The cluster Ba1 had a high relative abundance of the genera *Bacteroides* and *Faecalbacterium*. Cluster Bif showed a high prevalence of *Bifidobacterium*, while cluster Ba2 was characterized by a high relative abundance of *Bacteroides* and *Bifidobacterium*. The highest alpha diversity was observed for Sub, followed by Ba1, Bif, and finally, Ba2, which had the lowest diversity (*χ*^2^(3) = 90.206, *p* < 0.001; *χ*^2^(3) = 118.471, *p* < 0.001; *χ*^2^(3) = 99.074, *p* < 0.001, respectively) (Fig. 1B–D). Finally, beta diversity differed between the four clusters (Fig. 1E).

Parents of participants were predominantly white, highly educated (i.e., university degree or higher), and had a high annual income (i.e., $70,000–$99,9999 or higher) (Table 2, Table S1 provides an overview of all participants combined). Comparisons between the clusters revealed that the Sub and Ba1 clusters were predominantly white, while there were more non-white individuals (e.g., Chinese, South-East Asian, Latin American) within cluster Bif and Ba2 (F (3, 244) = 10.384, *p* < 0.001). In addition, cluster Ba2 was associated with a lower birth weight and gestational age (*F* (3, 243) = 4.751, *p* = 0.003; *F* (3, 243) = 4.721, *p* = 0.003, respectively), as well as an increased dietary intake of grain products (*F* (3, 197) = 2.966, *p* = 0.033).

**Table 2:** Sample characteristics

Maternal characteristics	Sub (*n* = 81)	Ba1 (*n* = 70)	Bif (*n* = 53)	Ba2 (*n* = 44)
Age (years), mean (SD, range)	32.5 (4.0, 20.3–40.7)	31.7 (4.1, 22.4–44.4)	33.1 (4.1, 24.3–42.8)	32.5 (4.0, 25.5–40.5)
Education, *N* (%)				
Completed high school diploma	6 (7.4%)	4 (5.7%)	3 (5.7%)	1 (2.3%)
Completed trade, technical diploma	13 (16.0%)	11 (15.7%)	12 (22.6%)	10 (22.7%)
Completed university degree	40 (49.4%)	45 (64.3%)	28 (52.8%)	24 (54.5%)
Completed post-graduate degree	22 (27.2%)	10 (14.3%)	10 (18.9%)	9 (20.5%)
Annual household income, *N* (%)^b^				
< $20,000	3 (3.7%)	0	1 (1.9%)	1 (2.3%)
$20, 000–$39,999	2 (2.5%)	4 (5.7%)	1 (1.9%)	1 (2.3%)
$40,000–$69,999	7 (8.6%)	5 (7.1%)	9 (17.0%)	6 (14.0%)
$70,000–$99,999	18 (22.2%)	20 (28.6%)	12 (22.6%)	7 (16.3%)
> $100,000	51 (63%)	41 (58.6%)	30 (56.6%)	28 (65.1%)
Ethnicity, *N* (%)				
White	**78 (96.3%)**	**66 (94.3%)**	**40 (75.5%)**	**30 (68.2%)**
Chinese	**0**	**0**	**4 (7.5%)**	**6 (13.6%)**
South-East Asian	**0**	**2 (2.9%)**	**4 (7.5%)**	**1 (2.3%)**
Latin America	**0**	**1 (1.4%)**	**3 (5.7%)**	**2 (4.5%)**
Other	**3 (3.7%)**	**1 (1.4%)**	**2 (3.8%)**	**5 (11.3%)**
Child characteristics				
Age (years), mean (SD, range)	4.35 (0.51, 3.06–4.95)	4.33 (0.45, 3.23–4.91)	4.47 (0.47, 3.05–5.00)	4.38 (0.47, 3.39–5.00)
Male, *N* (%)	45 (55.6%)	37 (52.9%)	25 (47.2%)	23 (52.3%)
Mode of delivery–vaginal, *N* (%)^b^	58 (71.6%)	52 (74.3%)	42 (79.2%)	35 (79.5%)
Gestational age (weeks), mean (SD, range)^b^	**39.6 (1.8, 33.7**–**41.9)**	**39.0 (1.5, 35.0**–**42.0)**	**39.3 (1.3, 35.6**–**41.4)**	**38.3 (2.5, 29.4**–**41.3)**
Birth weight (gram), mean (SD, range)^b^	**3451 (594, 2468**–**5210)**	**3334 (520, 2215**–**4649)**	**3383 (550, 2260**–**4904)**	**3052 (632, 1200**–**4185)**
Antibiotic exposure–yes, *N* (%)^c^	36 (44.4%)	28 (40.0%)	18 (34.0%)	16 (36.4%)
BASC-2, mean (SD, range)^b^				
Adaptive Skills (T score)	50.9 (8.4, 24–68)	53.2 (8.0, 32–67)	48.3 (7.8, 34–67)	49.7 (8.6, 30–68)
Internalizing (T score)	47.5 (8.5, 33–66)	48.2 (7.5, 30–66)	48.7 (8.4, 32–67)	48.4 (7.6, 3166)
Externalizing (T score)	49.9 (7.9, 38–74)	47.8 (8.5, 37–70)	49.4 (7.8, 36–66)	50.4 (7.7, 36–66)
Behavioral symptoms (T score)	49.4 (8.3, 35–73)	48.2 (6.9, 36–65)	50.9 (6.9, 35–66)	50.5 (8.3, 37–67)
Diet, mean (SD, range)^d^				
Diet quality score	3.70 (0.58, 1.95–4.93)	3.72 (0.75, 1.96–5.74)	3.66 (0.73, 2.29–5.71)	3.67 (0.68, 2.15–4.92)
Total energy intake (calories)	1540 (382, 614–2765)	1563 (397, 941–2736)	1644 (673, 924–4919)	1828 (1168, 761–5131)
Vegetable and fruit (gram)	491 (196, 164–1032)	498 (214, 164–1304)	480 (291, 174–1793)	507 (328, 93–1793)
Grain product (gram)	**142 (64, 12.7–356)**	**135 (52, 48–356)**	**149 (108, 44**–**660)**	**185 (115, 56**–**522)**
Meat and alternatives (gram)	108 (44, 20–249)	117 (46, 39–251)	123 (66, 13–370)	106 (76, 11–425)

^a^Data are presented as the number (percentage) of the non-missing values unless otherwise indicated (*n* = 248)

^b^Data missing from 1 participant

^c^Antibiotic exposure during the first 3 years of life

^d^Data missing from 47 participants. Variables that are different between groups are highlighted in bold.

### Differences in the fecal metabolome between clusters

**Fig. 2:**
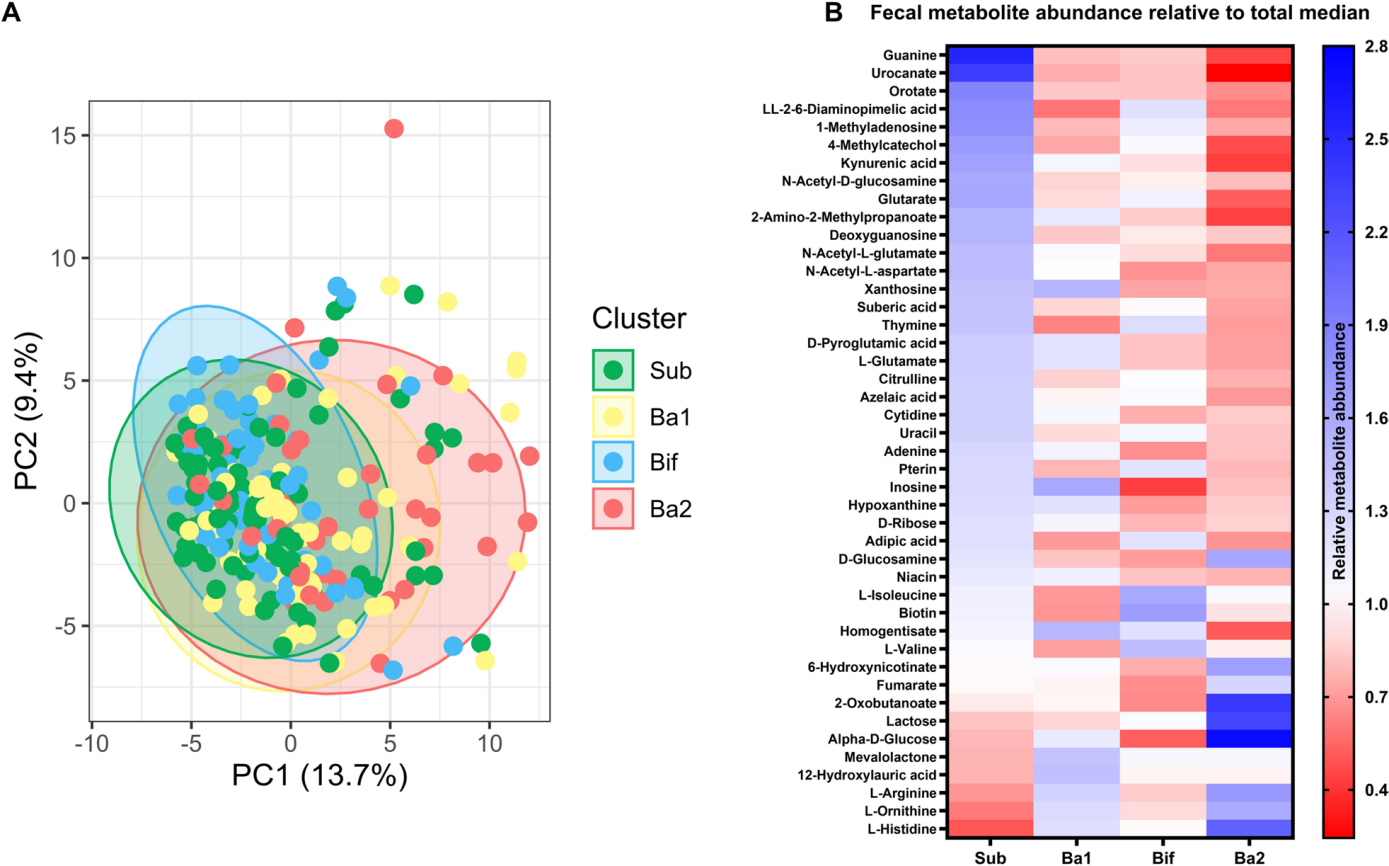
Stool metabolome differences between clusters. **A** Differences in beta diversity of the stool metabolome were assessed, where individual dots are datapoints. **B** 44 metabolites were found to be different using Kruskal-Wallis tests followed by a Benjamini-Hochberg correction, after which the relative differences of these metabolites were depicted in a heatmap. Total *n* = 248, cluster sub is *n* = 81, Ba1 is *n* = 70, Bif is *n* = 53, Ba2 is *n* = 44

Analysis of stool metabolome beta diversity between the groups revealed that cluster Ba2 had the most distinct metabolome compared to the rest of the clusters (*F*_1,245_ = 10.74, *p* < 0.001), while clusters Sub, Ba1, and Bif were still statistically different from all other clusters (*F*_1,245_ = 4.86, *p* = 0.008; *F*_1,245_ = 3.39, *p* = 0.035; *F*_1,245_ = 5.68, *p* = 0.004, respectively) (Fig. 2A). In particular, the covariates ethnicity and diet quality explained a significant portion of the variance in the stool metabolome (*F*_1,185_ = 2.12, *p* = 0.006; *F*_1,185_ = 2.07, *p* = 0.007) (Table S2). Analysis of the individual stool metabolites revealed that, of the 142 metabolites measured in the metabolomics assay, 44 metabolites were significantly different between the 4 clusters (Fig. 2B). Subsequent analyses grouping these metabolites into metabolic pathways using MetaboAnalyst detected a total of 50 metabolic pathways to which these 142 metabolites could be assigned. Cluster Sub had 16 metabolic pathways that were significantly differentially abundant, while cluster Ba1 had 7 differentially abundant metabolic pathways, cluster Bif had 12 differentially abundant metabolic pathways, and cluster Ba2 had 39 differentially abundant metabolic pathways (Fig. S3). It is also interesting to note that nucleotide levels were different between the clusters and that cluster Bif and Ba2 had lower levels of specific nucleotides (Fig. S4).

### Behavioral differences between the clusters

**Fig. 3:**
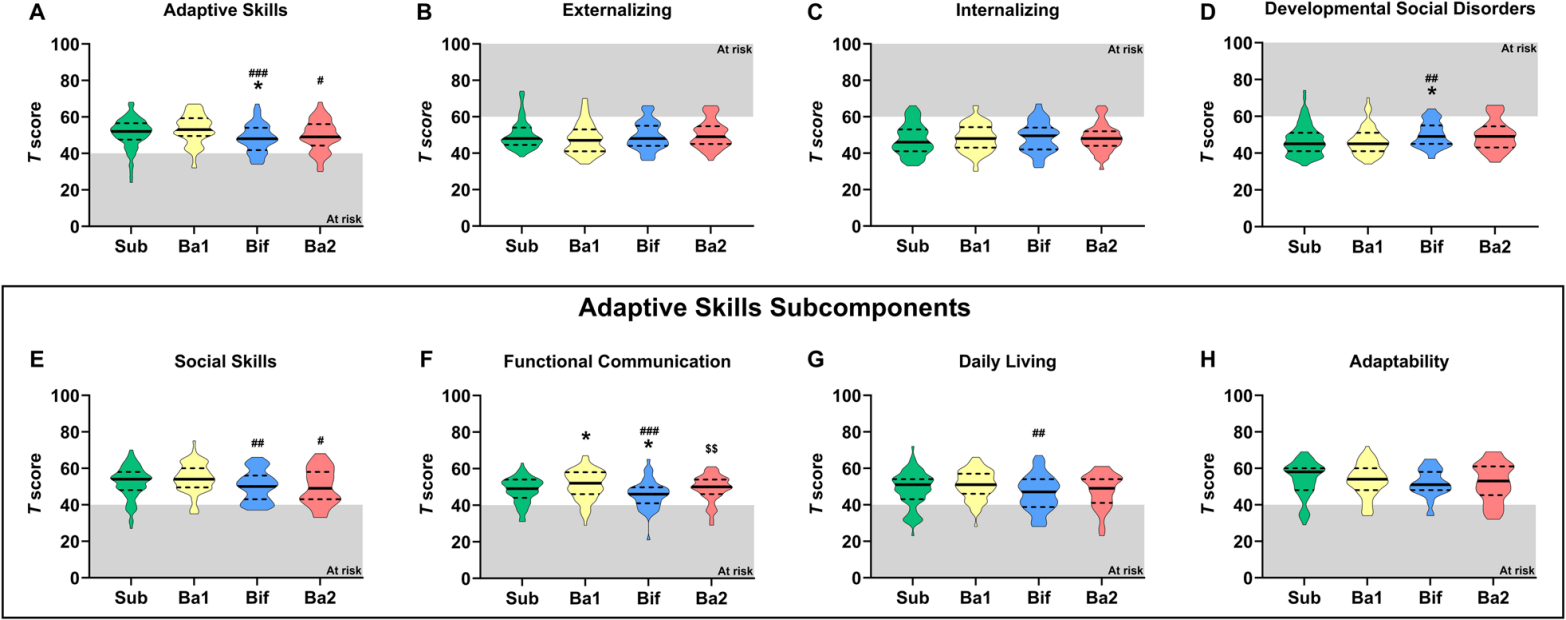
Behavioral differences between the four clusters. **A**–**D** The BASC-2 was used to assessed differences in Adaptive Skills, Externalizing, Internalizing and Developmental Social Disorders between the clusters. **E**–**H** The Adaptive Skills Subcomponents (i.e., Social Skills, Functional Communication, Daily Living, Adaptability) were subsequently compared between the clusters. Statistical differences were assessed using Kruskal-Wallis tests followed by Mann-Whitney tests. Data are depicted as violin plots where the middle line is the median and the dotted lines are quartiles. The grey zones depict the scores at which a participant would be “at risk” for an associated disorder. Total *n* = 248, cluster sub is *n* = 81, Ba1 is *n* = 70, Bif is n = 53, Ba2 is *n* = 44. For the statistical significance, *p < 0.05; **p < 0.01; ***p < 0.001, * indicates a statistical difference compared to the sub cluster, # compared to the Ba1 cluster, $ compared to the Bif cluster

There were differences in Adaptive Skill scores between clusters (χ^2^(3) = 13.799, *p* = 0.003) (Fig. 3A) whereas Internalizing, Externalizing, and Behavioural Symptom scores did not differ between clusters (Fig. 3B, C, Fig. S3). Specifically, clusters Bif and Ba2 were associated with reduced Adaptive Skill scores (Sub vs Bif: *U* = 1593.5, *p* = 0.018; Ba1 vs Bif: *U* = 1149.0, *p* < 0.001; Ba1 vs Ba2: *U* = 1152, *p* = 0.024). In addition, cluster Bif was associated with increased scores for Developmental Social Disorders (Sub vs Bif: *U*= 1583.5, *p* = 0.016; Ba1 vs Bif: *U* = 1308.5, *p* = 0.008) (Fig. 3D). No differences in Behavioral Symptoms scores were observed (Fig. S5). The differences in Adaptive Skill scores between clusters were explained by differences in the subcomponents including Social Skills, Functional Communication and Daily Living (*χ*^2^(3) = 10.120, *p* = 0.018; *χ*^2^(3) = 17.901, *p* < 0.001; *χ*^2^(3) = 8.670, *p* = 0.034, respectively), but not the subcomponent Adaptability (Fig. 3E–H). Bif and Ba2 were associated with reduced Social Skill score compared to Ba1 (Ba1 vs Bif: *U* = 1316.0, *p* = 0.006; Ba1 vs Ba2: *U* = 1115.0, *p* = 0.013). In addition, Ba1 was associated with increased Functional Communication scores compared to Sub (*U* = 2187.5, *p* = 0.015), while Bif was associated with reduced Functional Communication scores compared to all groups (Sub: *U* = 1617.0, *p* = 0.016; Ba1: U = 1115.0, *p* < 0.001; Ba2: *U* = 782.5, *p* = 0.005). Finally, Bif was associated with reduced Daily Living scores compared to Ba1 (*U* = 1289, *p* = 0.004). As there were differences in gestational age at birth, birth weight, ethnicity, and grain intake, we used an ANCOVA to control for these covariates (Tables S3, S4, S5, S6, S7). Controlling for covariates reduced the mean difference in Developmental Social Disorder Scores between the Sub and Bif clusters by 1.14 points (mean difference without covariates = 3.00, *p* = 0.033; mean difference with covariates = 1.87, *p* = 0.256). Controlling for individual covariates revealed that this was predominantly driven by ethnicity and grain intake (mean difference when controlling for only ethnicity = 2.42; gestational age at birth = 2.91; birth weight = 2.93; grain intake = 2.54). Similarly, controlling for covariates reduced the mean difference in Developmental Social Disorder Scores between the Ba1 and Bif clusters by 1.00 points (mean difference without covariates = 3.40, *p* = 0.019; mean difference with covariates = 2.40, *p* = 0.148). Controlling for individual covariates revealed that this was predominantly driven by ethnicity and grain intake (mean difference when controlling for only ethnicity = 2.87; gestational age at birth = 3.50; birth weight = 3.52; grain intake = 2.71). Controlling for covariates reduced the mean difference in Functional Communication scores between the Sub and Bif clusters by 0.90 points (mean difference without covariates = 2.38, *p* = 0.076; mean difference with covariates = 1.47, *p* = 0.371). Controlling for individual covariates revealed that this was predominantly driven by grain intake (mean difference when controlling for only ethnicity = 2.17; gestational age at birth = 2.33; birth weight = 2.34; grain intake = 1.56). Similarly, controlling for covariates reduced the mean difference in Functional Communication scores between the Ba2 and Bif clusters by 0.83 points (mean difference without covariates = 3.32, *p* = 0.032; mean difference with covariates = 2.49, *p* = 0.195). Controlling for individual covariates revealed that this was predominantly driven by grain intake (mean difference when controlling for only ethnicity = 3.39; gestational age at birth = 3.41; birth weight = 3.68; grain intake = 2.32).

### Cluster-dependent associations between galactose metabolism and daily living

**Fig. 4:**
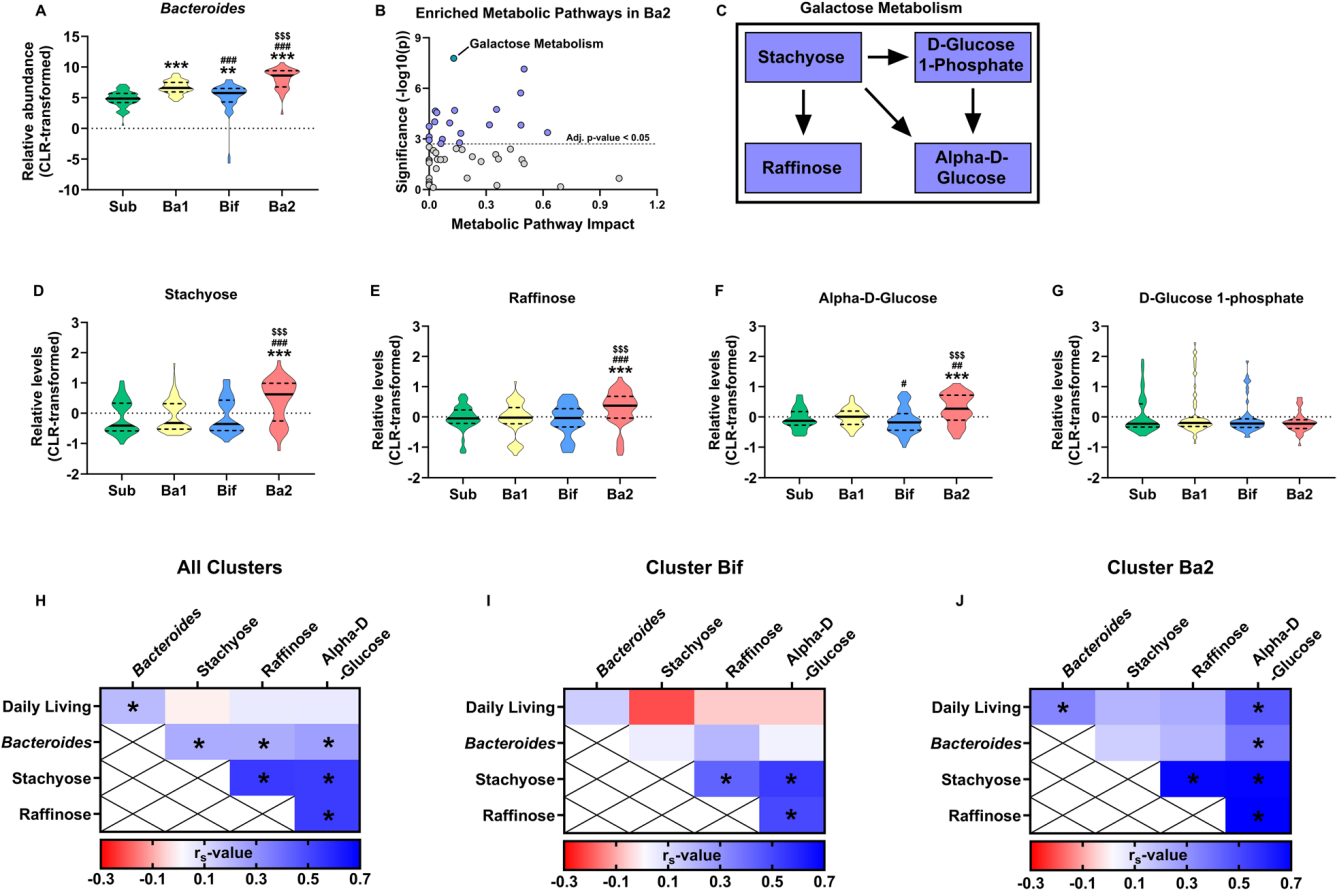
Cluster-dependent associations between Daily Living scores and stool galactose metabolism. **A** Relative *Bacteroides* abundances were compared between the clusters. **B**, **C** MetaboAnalyst was used to identify differentially abundant metabolic pathways in cluster Ba2, where galactose metabolism was observed as the most significant pathway. **D**–**G** The individual metabolites of this pathway were then compared between the clusters, which were stachyose, raffinose, alpha-D-glucose, and D-glucose-1-phosphate. **H** Daily Living scores, relative *Bacteroides* abundances, and identified metabolites were subsequently correlated with each other using data from all participants, **I** only those part of cluster Bif, **K** or only those part of cluster Ba2. For **A** and **D**–**J** statistical differences were assessed using Kruskal-Wallis tests followed by Mann-Whitney tests. Data are depicted as violin plots where the middle line is the median and the dotted lines are quartiles. For the statistical significance of **A**–**H** *p < 0.05; **p < 0.01; ***p < 0.001, * indicates a statistical difference compared to the sub cluster, # compared to the Ba1 cluster, $ compared to the Bif cluster. For **I**–**K** statistical significance was depicted as *p < 0.05. Total *n* = 248, cluster sub is *n* = 81, Ba1 is *n* = 70, Bif is *n* = 53, Ba2 is *n* = 44

We sought to identify associations between child behavior and the gut microbiota/metabolome that were cluster-dependent. As cluster Ba2 metabolome was most distinct compared to the other clusters, we subsequently identified the most altered metabolic pathway using MetabAnalyst, which was Galactose Metabolism (Adj. *p* < 0.001, impact = 0.129) (Fig. 4A–C). In this pathway, cluster Ba2 was enriched in the metabolites stachyose, raffinose and alpha-D-glucose (χ^2^(3) = 22.554, *p* < 0.001; *χ*^2^(3) = 18.605, *p* < 0.001; *χ*^2^(3) = 22.592, *p* < 0.001), but not D-glucose-1-phosphate, compared to the three other clusters (Fig. 4D–G). Finally, we correlated Daily Living scores, relative *Bacteroides* abundance, and the three galactose metabolism metabolites with each other in all the participants, only within the Bif cluster, or only within the Ba2 cluster (Fig. 4H–J). There was a significant association between Daily Living scores with relative *Bacteroides* abundance and alpha-D-glucose levels within the Ba2 cluster (*r*_s_ = 0.189, *p* = 0.003; *r*_s_ = 0.456, *p* = 0.002, respectively). Adjusting for ethnicity, gestational age at birth, birth weight, and child grain intake reduced the effect size of the association between alpha-D-glucose levels and Daily Living Scores (*r* = 0.311, *p* = 0.107). Controlling for individual covariates revealed that this was predominantly driven by grain intake (controlling for only ethnicity: *r* = 0.365, *p* = 0.016; gestational age at birth: *r* = 0.332, *p* = 0.032; birth weight: *r* = 0.330, *p* = 0.031; grain intake: *r* = 0.310, *p* = 0.090). In addition, relative *Bacteroides* abundance and alpha-D-glucose levels correlated within the Ba2 cluster (*r*_s_ = 0.333, *p* = 0.029). These correlations were absent in the Bif cluster. It is also noteworthy that the effect sizes for these associations were greater in the Ba2 cluster, compared to the entire population (Daily Living scores vs relative *Bacteroides* within the entire population: *r*_s_ = 0.187, *p* = 0.003; within the Ba2 cluster: *r*_s_ = 0.333, *p* = 0.029).

### Cluster-dependent associations between the histidine to urocanate breakdown and social skills

**Fig. 5:**
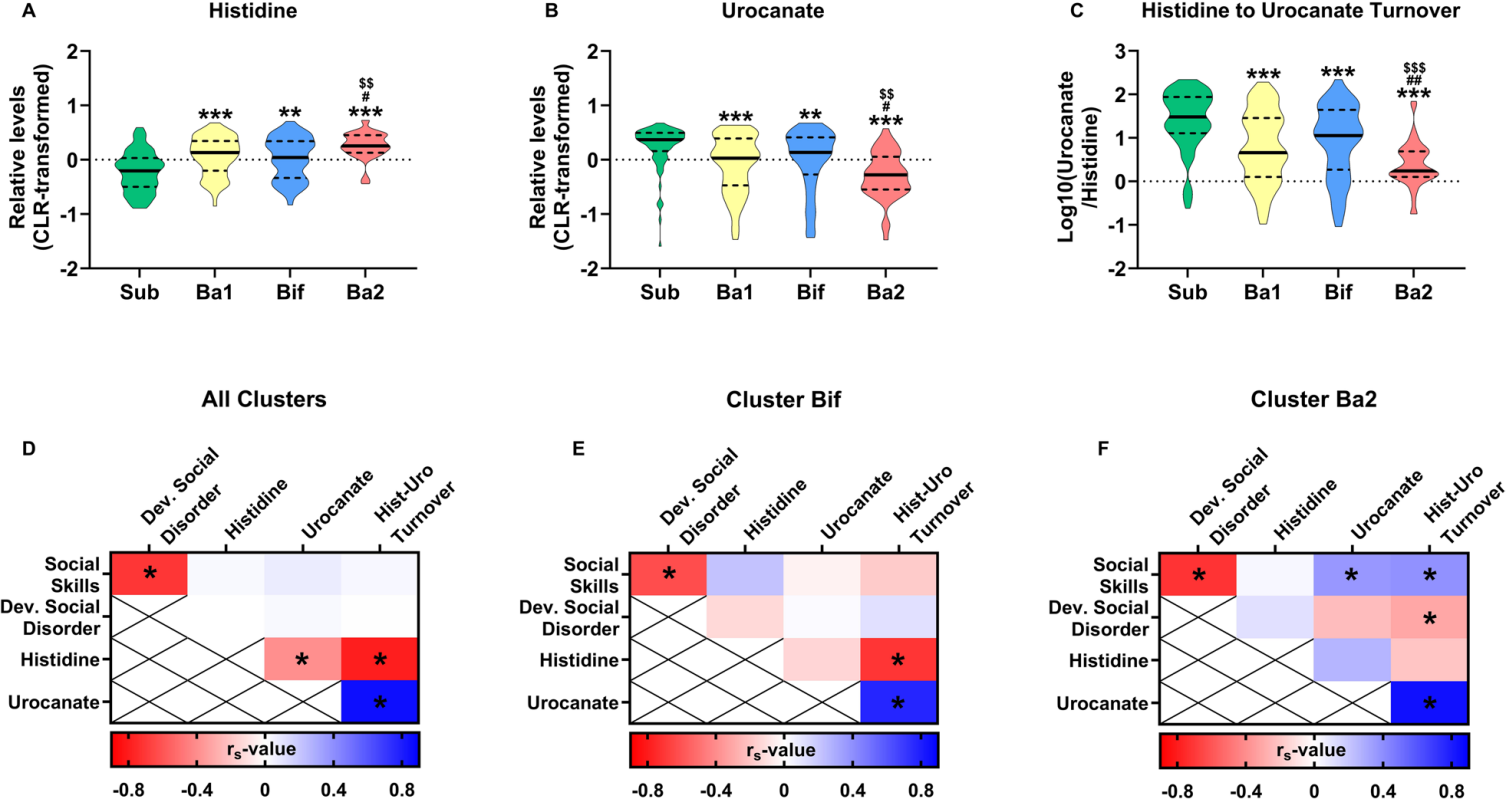
Cluster-dependent associations between Social Skills and stool histidine to urocanate turnover. **A**–**C** Relative levels of stool histidine and urocanate were quantified, after which the turnover rate was calculated by dividing urocanate by histidine levels (depicted as Log10 ratio in **C**). **D** Social Skill scores, Developmental Social Disorder scores, and identified metabolites were subsequently correlated with each other using data from all participants, **E** only those part of cluster Bif, F or only those part of cluster Ba2. For **A**–**C** statistical differences were assessed using Kruskal-Wallis tests followed by Mann-Whitney tests. Data are depicted as violin plots where the middle line is the median and the dotted lines are quartiles. For the statistical significance of **A**–**C** *p < 0.05; **p < 0.01; ***p < 0.001, * indicates a statistical difference compared to the sub cluster, # compared to the Ba1 cluster, $ compared to the Bif cluster. For **D**–**F** statistical significance was depicted as *p < 0.05. Total *n* = 248, cluster sub is *n* = 81, Ba1 is *n* = 70, Bif is *n* = 53, Ba2 is *n* = 44

Manual pathway curation of significantly altered metabolites between clusters using the KEGG database revealed that 2 of these metabolites were directly connected, where histidine is metabolized into urocanate [53]. The metabolomics analyses revealed that stool histidine levels were increased in cluster Ba2 (*χ*^2^(3) = 39.113, *p* < 0.001), while urocanate levels were reduced (*χ*^2^(3) = 36.771, *p* < 0.001) (Fig. 5A, B). This is particularly noteworthy because the primary source of urocanate is the breakdown of histidine [53]. Calculating the turnover rate of histidine to urocanate subsequently revealed that the turnover of histidine to urocanate was lowest for cluster Ba2 (*χ*^2^(3) = 52.562, *p* < 0.001; the medians of non-normalized rates were Sub = 30.0, Ba1 = 4.5, Bif = 11.3, Ba2 = 1.8) (Fig. 5C). We subsequently correlated histidine, urocanate and their turnover rate with Social Skill scores and Developmental Social Disorder scores to investigate if there was a (cluster-dependent) association between the stool metabolome and social behavior (Fig. 5D–F). Indeed, there was a significant association between Urocanate and Histidine-Urocanate turnover rates with Social Skill scores (*r*_s_ = 0.365, *p* = 0.015; *r*_s_ = 0.388, *p* = 0.009, respectively), while Histidine-Urocanate turnover rates additionally correlated with Developmental Social Disorder scores (*r*_s_ = − 0.316, *p* = 0.037). These associations were absent in the sample as a whole or in other clusters. Adjusting for ethnicity, gestational age at birth, birth weight and child grain intake did not affect the association between urocanate-histidine turnover with Social Skill scores (*r* = 0.344, *p* = 0.073), but did modestly reduce the effect size of the association between urocanate-histidine turnover with Developmental Social Disorder scores (*r* = − 0.271, *p* = 0.163). Controlling for individual covariates revealed that this was predominantly driven by ethnicity and grain intake (controlling for only ethnicity: *r* = − 0.272, *p* = 0.078; gestational age at birth: *r* = − 0.294, *p* = 0.059; birth weight: *r* = − 0.312, *p* = 0.041; grain intake: *r* = − 0.292, *p* = 0.111).

Next, we investigated if the gut microbiota played a role in the differential turnover of histidine to urocanate between the clusters. To this end, we correlated gut microbial taxa with histidine-urocanate turnover rates in the entire population, cluster Bif, and cluster Ba2 (Fig. S6). Of the 69 investigated taxa, 46 correlated with histidine to urocanate turnover in the entire population (min *r*_s_ = − 0.489, max *r*_s_ = 0.466), 19 when only investigating the Bif cluster participants (min *r*_s_ = − 0.442, max *r*_s_ = 0.439), and 3 when only investigating the Ba2 cluster participants (min *r*_s_ = − 0.316, max *r*_s_ = 0.339). These data suggest that the gut microbiota may be less involved in histidine-to-urocanate turnover for children with a Ba2 cluster, compared to the other clusters.

Finally, we investigated whether the relationship between Social Skills and Developmental Social Disorders scores with urocanate levels and histidine-urocanate turnover were also present in children with reduced histidine-urocanate turnover rates, or whether these relationships were specific to children in cluster Ba2. After selecting all participants (*n* = 64) with a histidine-urocanate turnover rate > 2 (the median turnover rate for cluster Ba2 was 1.75), there was no correlation between Social Skills and Developmental Social Disorders scores with either urocanate or histidine-urocanate turnover rates (Fig. S7). Lastly, we investigated whether children with lower Social Skills or Developmental Social Disorders scores had altered levels of urocanate and histidine-urocanate turnover rates. However, there were no differences in urocanate levels or histidine-urocanate turnover rates between children who had “at risk” scores for Social Skills or Developmental Social Disorders (Fig. S8). These findings indicate that the relationship between histidine-urocanate turnover and Social Skill scores is specific to cluster Ba2, as opposed to individuals with either low histidine-urocanate turnover rates or Social Skill scores. And that increasing histidine-urocanate turnover for individuals with low histidine-urocanate turnover or Social Skill scores may less likely to improve Social Skill scores, compared to increasing histidine-urocanate turnover for individuals with a Ba2 gut microbiota cluster.

## Discussion

These data reveal novel insights into how the composition of the gut microbiota and its metabolites are associated with child behavior during early life; therefore, expanding our knowledge of the microbiota-gut-brain axis. Cluster Ba2 showed a distinct stool metabolome and unique cluster-dependent associations between histidine-urocanate metabolism and Social Skills, as well as galactose metabolism and Daily Living scores.

Our cluster analysis in preschool children revealed four distinct clusters characterized by high relative abundances of *Bacteroides*, *Subdoligranulum*, and *Bifidobacterium*, while previous analyses in adults have identified 3–4 clusters characterized by high relative abundances of *Bacteroides*, *Prevotella*, and *Ruminococcaceae* [16, 22, 23]. These differences in cluster composition could be related to age, considering that the microbiota undergoes change throughout childhood and even into adolescence to a lesser extent [55]. Indeed, a key bacterial taxon within the cluster groupings of our analysis is *Bifidobacterium*, which was reported to be a key taxon for cluster groupings for infants during the first month of life [56]. In addition, another study reported a *Bifidobacterium*-enriched cluster in children at 6–9 years of age [25]. The prevalence of the *Bifidobacterium*-dominant cluster might be related to the relatively high abundance of *Bifidobacterium* species in infants and children, which gradually decreases until adulthood [55, 57, 58], likely explaining why *Bifidobacterium*-predominant clusters are frequently reported in studies investigating the child gut microbiota, but not in adults. It is also interesting to note that the cluster characterized by high *Subdoligranulum* abundance (Sub) had the highest prevalence of *Rumminococcus*_1 and *Rumminococcus*_2, indicating that this cluster might closely resemble the *Ruminococcaceae* cluster reported in adults. Finally, the cluster Ba2 observed in our dataset somewhat resembles the B2 cluster observed in adults, as it is characterized by low relative abundances of *Faecalibacterium* [17]. Overall, these data suggest that the observed clusters in this study may resemble the clusters observed in adults, albeit still in a developing phase, as gut microbiota clusters start to reach a stable phase between 3 and 4 years of age [59]. As such, more research is warranted into the future developmental trajectories of gut microbiota clusters in developing children.

Cluster Bif was associated with increased scores for Developmental Social Disorders and reduced Adaptive Skills scores—specifically the subcomponents Social Skills, Functional Communication, and Daily Living. Even though we are unaware of any studies linking *Bifidobacterium* abundances to child behavioral outcomes at preschool age, some studies show associations between *Bifidobacterium* abundances and temperament during the first year of life [24, 60–62]. Those studies suggest that elevated *Bifidobacterium* abundances are associated with temperament among infants, but because of the highly dynamic nature of the gut microbiota during infancy, it is not clear how those findings might relate to behavioral outcomes at preschool age. Taken together with the previous studies of infancy and the fact that the Bif cluster was associated with reduced Adaptive Skill scores and an increase in Developmental Social Disorder scores suggests that the link between behavior and *Bifidobacterium* requires further investigation during childhood. Our data also show that cluster Ba2 was associated with reduced Adaptive Skill scores—specifically the subcomponents of Social Skills and Functional Communication. A *Bacteroides*-dominant cluster at 2.5 months old was associated with lower regulation scores on the IBQ-R [24]. In addition, a *Bacteroides*-dominant cluster at 1 year of age was associated with increased cognitive scores [63, 64]. Altogether, our finding showing that the Bif and Ba2 clusters are associated with poorer Adaptive Skills is not consistently observed across the literature, but these discrepancies may be explained by the age at which the gut microbiota was measured. Previous studies have predominantly focussed on the first 2 years of life, while our study investigated the gut microbiota at 3–4 years of life, which is important because the gut microbiota is believed to achieve an adult-like composition around 3 years of age [55].

Cluster Ba2 had a more distinct stool metabolome compared to the other clusters. Our analyses revealed two metabolic processes that were associated with child behavioral outcomes, based on the MetabolAnalyst results and manual identification of metabolites that are directly metabolized into each other. These two metabolic processes were histidine to urocanate metabolism and galactose metabolism. In addition, significantly fewer bacterial taxa correlated with histidine-urocanate turnover rates for cluster Ba2. Similarly, the introduction of a specific pathogen-free microbiota to mice without any microbiota (i.e., germ-free) reduces fecal histidine levels and increases urocanate levels [65]. These data may suggest a reduced involvement of the gut microbiota in histidine-to-urocanate metabolism, which may ultimately result in the deficit of systemic urocanate levels and its downstream metabolites. It is also noteworthy that histidine can be metabolized into histamine through histidine decarboxylase [66], where histamine plays a key role in immune system functioning and has been linked to neuronal development [67], even though our study did not quantify histamine. As for galactose metabolism, the Ba2 cluster-specific association between *Bacteroides* relative abundances and stachyose, raffinose, and alpha-D-glucose may suggest that these saccharides could be a substrate for taxa within the *Bacteroides* genus in individuals who have a Ba2 gut microbiota cluster specifically. In tandem, cluster Ba2 was associated with an elevated grain intake and controlling for grain intake showed a modest reduction in the effect-size of the correlation between alpha-D-glucose levels and Daily Living Scores, which is important because stachyose (a precursor of alpha-D-glucose) is present in grains. Other studies have also reported that altered carbohydrate metabolism is a unique feature of cluster Ba2 in adults [68, 69]. Finally, our metabolomics analyses revealed differences in nucleotide levels between the clusters, where Bif and Ba2 clusters showed lower levels. This could be linked to differences in stool bacterial load (i.e., the number of bacteria present per gram stool), as Ba2 has been associated with a lower bacterial load [18, 22]. Future studies should include the analysis of bacterial load to normalize the relative gut microbial abundances. Overall, some of the metabolomic differences characterized within our results are largely in line with previous reports focussing on cluster analyses in adults.

The concept of gut microbiota clusters was initially highly debated as clusters are more continuous, rather than distinct gut microbiota compositions [70, 71]. Consequently, an individual’s gut microbiota may switch clusters over time [70, 71]. Nonetheless, subsequent reports have shown that the overall cluster groupings and their functional metabolic capacity are replicable even between cohorts and different populations [68, 72]. In addition, cluster Ba2 has been repeatedly associated with negative health outcomes in adults, such as increased systemic inflammation, lower cardiac vagal function, reduced quality of life scores, and increased odds for obesity, type 2 diabetes, Crohn’s disease, and depression [18–23]. A cluster enriched in *Faecalbacterium* and *Bacteroides* abundance has been linked to reduced Receptive Language and Expressive Language scores assessed using the Mullen Scales of Early Learning at 2 years of age [73]. In tandem, our data reveals that cluster Ba2 is associated with reduced Adaptive Skill and Social Skill scores. Similarly, our analyses did not detect any differences in internalizing and externalizing behaviors, which is in line with previous work [8]. Overall, even though cluster-based approaches for understanding the relationship between the gut microbiota and host physiology/behavior have clear limitations, cluster-related findings tend to be consistent across populations, where cluster Ba2 is the most consistently associated with unfavorable health outcomes. If these unfavorable mental health outcomes associated with cluster Ba2 are consistent across different ages, then interventions for improving these behavioral outcomes in individuals with a Ba2 gut microbiome during childhood may be associated with improved mental health outcomes at a later age, as these behavioral outcomes have been shown to predict mental health outcomes in later life [14, 74].

### Strengths and limitations

A limitation of this study is the lack of repeated child stool sampling, as an individual's gut microbiota may switch clusters over time [70, 71]. This study also used 16S rRNA short-read sequencing, which results in a lower genomic resolution compared to whole genome sequencing. In addition, even though clustering methods are a useful tool to compress high-dimensional data, they can also lead to data loss. As such, more gut microbiota-wide interaction analyses are warranted to understand the conditional effects of individual gut microbial taxa on host physiology and behavior. Another limitation is that participants were predominantly White and had a relatively high socioeconomic status, which reduces the generalizability of the study results. A major strength of this study is the assessment of the child’s fecal metabolome, which is important because one of the primary ways in which the gut microbiota affects physiology is through the metabolites it produces [75].

## Conclusions

Our data analyses reveal novel relationships between histidine-urocanate turnover with Social Skills and Developmental Social Disorder scores, as well as galactose metabolism and Daily Living scores, both of which are only present in cluster Ba2. These results provide novel insights into conditional gut microbiome effects on microbial metabolism and child behavioral outcomes. Interestingly, baseline gut microbiome compositions may provide insights into treatment efficacy [10–13], as clusters pre-intervention predict gut microbiome changes in response to nutritional interventions [76]. In addition, the *Prevotella*-to-*Bacteroides* ratio has been shown to predict body weight and fat loss changes induced by dietary interventions [77–79]. Furthermore, type 2 diabetes patients with a high abundance of *Bacteroides* show a larger improvement in metabolic parameters following treatment with the type 2 diabetes medication Acarbose, compared to patients with high *Prevotella* abundance [80]. As such, this emerging data on the conditional effects of the gut microbiota or gut microbial clusters on interventions, physiology (e.g., stool metabolome), and health outcomes (e.g., behavior), may potentially lead to useful tools for personalizing microbiome-targeted interventions for improving health outcomes.

### Supplementary Information

**Additional file 1:** **Figure S1.** Gut microbiota clustering using Dirichlet multinomial mixtures. **Figure S2.** Flowchart of data analysis approach. **Figure S3.** Differences in stool metabolic pathways between the four clusters. **Figure S4.** Differences in stool nucleotide levels between the four clusters. **Figure S5.** Behavioral Symptom scores between the four clusters. **Figure S6.** Number of microbial taxa that correlate with stool histidine to urocanate turnover. **Figure S7.** Relationship between Social Skills and urocanate metabolism for participants with low histidine-urocanate turnover rates. **Figure S8.** Levels of stool urocanate and histidine-urocanate turnover between children with “at risk” and “not at risk” scores for Social Skills and Developmental Social Disorders. **Table S1.** Sample characteristics of all combined participants. **Table S2.** PERMANOVA results for stool metabolome beta diversity. Individual independent effects are depicted. **Table S3.** ANCOVA Results for Adaptive Skill Scores While Correction for Gestational Age at Birth, Birth Weight, Ethnicity and Child Grain Intake. **Table S4.** ANCOVA Results for Developmental Social Disorder Scores While Correction for Gestational Age at Birth, Birth Weight, Ethnicity and Child Grain Intake. **Table S5.** ANCOVA Results for Social Skill Scores While Correction for Gestational Age at Birth, Birth Weight, Ethnicity and Child Grain Intake. **Table S6.** ANCOVA Results for Functional Communication Scores While Correction for Gestational Age at Birth, Birth Weight, Ethnicity and Child Grain Intake. **Table S7.** ANCOVA Results for Daily Living Scores While Correction for Gestational Age at Birth, Birth Weight, Ethnicity and Child Grain Intake.

## Data Availability

Raw gut microbiota reads have been deposited to the Sequence Read Archive under accession number PRJNA1031659.

## Abbreviations

ANOVA: One-way analysis of variance
APrON: Alberta Pregnancy Outcomes and Nutrition
Ba1: Gut microbiota cluster *Bacteroides* 1
Ba2: Gut microbiota cluster *Bacteroides* 2
BASC-2: Behavioral Assessment System for Children
Bif: Gut microbiota cluster *Bifidobacterium*
CBCL: Child Behavior Checklist
FDR: False-discovery rate
FFQ: Food Frequency Questionnaire
KEGG: Kyoto encyclopedia of genes and genomes
Sub: Gut microbiota cluster *Subdoligranulum*

## References

[1] Cowan CSM, Dinan TG, Cryan JF (2020). Annual research review: critical windows - the microbiota-gut-brain axis in neurocognitive development. J Child Psychol Psychiatry.

[2] Cryan JF, O'Riordan KJ, Cowan CSM, Sandhu KV, Bastiaanssen TFS, Boehme M, Codagnone MG, Cussotto S, Fulling C, Golubeva AV (2019). The microbiota-gut-brain axis. Physiol Rev.

[3] van de Wouw M, Wang Y, Workentine ML, Vaghef-Mehrabani E, Dewey D, Reimer RA, Tomfohr-Madsen L, Giesbrecht GF (2022). Associations between the gut microbiota and internalizing behaviors in preschool children. Psychosom Med.

[4] Loughman A, Ponsonby AL, O'Hely M, Symeonides C, Collier F, Tang MLK, Carlin J, Ranganathan S, Allen K, Pezic A (2020). Gut microbiota composition during infancy and subsequent behavioural outcomes. EBioMedicine.

[5] Laue HE, Karagas MR, Coker MO, Bellinger DC, Baker ER, Korrick SA, Madan JC (2022). Sex-specific relationships of the infant microbiome and early-childhood behavioral outcomes. Pediatr Res.

[6] McMath AL, Aguilar-Lopez M, Cannavale CN, Khan NA, Donovan SM (2023). A systematic review on the impact of gastrointestinal microbiota composition and function on cognition in healthy infants and children. Front Neurosci.

[7] Flannery JE, Stagaman K, Burns AR, Hickey RJ, Roos LE, Giuliano RJ, Fisher PA, Sharpton TJ (2020). Gut feelings begin in childhood: the gut metagenome correlates with early environment, caregiving, and behavior. mBio.

[8] Ou Y, Belzer C, Smidt H, de Weerth C (2022). Development of the gut microbiota in healthy children in the first ten years of life: associations with internalizing and externalizing behavior. Gut Microbes.

[9] Louca S, Polz MF, Mazel F, Albright MBN, Huber JA, O'Connor MI, Ackermann M, Hahn AS, Srivastava DS, Crowe SA (2018). Function and functional redundancy in microbial systems. Nat Ecol Evol.

[10] Gibbons SM, Gurry T, Lampe JW, Chakrabarti A, Dam V, Everard A, Goas A, Gross G, Kleerebezem M, Lane J (2022). Perspective: leveraging the gut microbiota to predict personalized responses to dietary, prebiotic, and probiotic interventions. Adv Nutr.

[11] Adan RAH, van der Beek EM, Buitelaar JK, Cryan JF, Hebebrand J, Higgs S, Schellekens H, Dickson SL (2019). Nutritional psychiatry: towards improving mental health by what you eat. Eur Neuropsychopharmacol.

[12] Zeevi D, Korem T, Zmora N, Israeli D, Rothschild D, Weinberger A, Ben-Yacov O, Lador D, Avnit-Sagi T, Lotan-Pompan M (2015). Personalized nutrition by prediction of glycemic responses. Cell.

[13] Kolodziejczyk AA, Zheng D, Elinav E (2019). Diet-microbiota interactions and personalized nutrition. Nat Rev Microbiol.

[14] Basten M, Tiemeier H, Althoff RR, van de Schoot R, Jaddoe VW, Hofman A, Hudziak JJ, Verhulst FC, van der Ende J (2016). The stability of problem behavior across the preschool years: an empirical approach in the general population. J Abnorm Child Psychol.

[15] Gleason MM, Goldson E, Yogman MW (2016). Council On Early C, Committee On Psychosocial Aspects Of C, Family H, Section On D, Behavioral P: Addressing Early Childhood Emotional and Behavioral Problems. Pediatrics.

[16] Arumugam M, Raes J, Pelletier E, Le Paslier D, Yamada T, Mende DR, Fernandes GR, Tap J, Bruls T, Batto JM (2011). Enterotypes of the human gut microbiome. Nature.

[17] Bresser LRF, de Goffau MC, Levin E, Nieuwdorp M (2022). Gut microbiota in nutrition and health with a special focus on specific bacterial clusters. Cells.

[18] Vieira-Silva S, Falony G, Belda E, Nielsen T, Aron-Wisnewsky J, Chakaroun R, Forslund SK, Assmann K, Valles-Colomer M, Nguyen TTD (2020). Statin therapy is associated with lower prevalence of gut microbiota dysbiosis. Nature.

[19] Vieira-Silva S, Sabino J, Valles-Colomer M, Falony G, Kathagen G, Caenepeel C, Cleynen I, van der Merwe S, Vermeire S, Raes J (2019). Quantitative microbiome profiling disentangles inflammation- and bile duct obstruction-associated microbiota alterations across PSC/IBD diagnoses. Nat Microbiol.

[20] Morkl S, Oberascher A, Tatschl JM, Lackner S, Bastiaanssen TFS, Butler MI, Moser M, Fruhwirth M, Mangge H, Cryan JF (2022). Cardiac vagal activity is associated with gut-microbiome patterns in women-An exploratory pilot study. Dialogues Clin Neurosci.

[21] Wang J, Li W, Wang C, Wang L, He T, Hu H, Song J, Cui C, Qiao J, Qing L (2020). Enterotype bacteroides is associated with a high risk in patients with diabetes: a pilot study. J Diabetes Res.

[22] Vandeputte D, Kathagen G, D'Hoe K, Vieira-Silva S, Valles-Colomer M, Sabino J, Wang J, Tito RY, De Commer L, Darzi Y (2017). Quantitative microbiome profiling links gut community variation to microbial load. Nature.

[23] Valles-Colomer M, Falony G, Darzi Y, Tigchelaar EF, Wang J, Tito RY, Schiweck C, Kurilshikov A, Joossens M, Wijmenga C (2019). The neuroactive potential of the human gut microbiota in quality of life and depression. Nat Microbiol.

[24] Aatsinki AK, Lahti L, Uusitupa HM, Munukka E, Keskitalo A, Nolvi S, O'Mahony S, Pietila S, Elo LL, Eerola E (2019). Gut microbiota composition is associated with temperament traits in infants. Brain Behav Immun.

[25] Zhong H, Penders J, Shi Z, Ren H, Cai K, Fang C, Ding Q, Thijs C, Blaak EE, Stehouwer CDA (2019). Impact of early events and lifestyle on the gut microbiota and metabolic phenotypes in young school-age children. Microbiome.

[26] Johnson AJ, Vangay P, Al-Ghalith GA, Hillmann BM, Ward TL, Shields-Cutler RR, Kim AD, Shmagel AK, Syed AN, Personalized Microbiome Class S, et al: Daily sampling reveals personalized diet-microbiome associations in humans. Cell Host Microbe 2019, 25:789-802 e785. 10.1016/j.chom.2019.05.005 31194939

[27] Javdan B, Lopez JG, Chankhamjon P, Lee YJ, Hull R, Wu Q, Wang X, Chatterjee S, Donia MS (2020). Personalized mapping of drug metabolism by the human gut microbiome. Cell.

[28] Kaplan BJ, Giesbrecht GF, Leung BM, Field CJ, Dewey D, Bell RC, Manca DP, O'Beirne M, Johnston DW, Pop VJ (2014). The Alberta Pregnancy Outcomes and Nutrition (APrON) cohort study: rationale and methods. Matern Child Nutr.

[29] Letourneau N, Aghajafari F, Bell RC, Deane AJ, Dewey D, Field C, Giesbrecht G, Kaplan B, Leung B, Ntanda H, Team APS (2022). The Alberta Pregnancy Outcomes and Nutrition (APrON) longitudinal study: cohort profile and key findings from the first three years. BMJ Open.

[30] Reynolds CR, Kamphaus RW (2015). Behavior assessment system for children.

[31] Illumina: Preparing 16S ribosomal RNA gene amplicons for the Illumina MiSeq system. Illumina technical note 2011.

[32] Ewels P, Magnusson M, Lundin S, Kaller M (2016). MultiQC: summarize analysis results for multiple tools and samples in a single report. Bioinformatics.

[33] Martin M (2011). Cutadapt removes adapter sequences from high-throughput sequencing reads. EMBnet J.

[34] Callahan BJ, McMurdie PJ, Rosen MJ, Han AW, Johnson AJ, Holmes SP (2016). DADA2: High-resolution sample inference from Illumina amplicon data. Nat Methods.

[35] R: A language and environment for statistical computing

[36] Wang Q, Garrity GM, Tiedje JM, Cole JR (2007). Naive Bayesian classifier for rapid assignment of rRNA sequences into the new bacterial taxonomy. Appl Environ Microbiol.

[37] Callahan B: Silva taxonomic training data formatted for DADA2 (Silva version 132). 2018.

[38] Price MN, Dehal PS, Arkin AP (2010). FastTree 2–approximately maximum-likelihood trees for large alignments. PLoS One.

[39] Nawrocki EP: Structural RNA homology search and alignment using covariance models. Washington University School of Medicine, 2009.

[40] Chen L, Reeve J, Zhang L, Huang S, Wang X, Chen J (2018). GMPR: A robust normalization method for zero-inflated count data with application to microbiome sequencing data. PeerJ.

[41] Love MI, Huber W, Anders S (2014). Moderated estimation of fold change and dispersion for RNA-seq data with DESeq2. Genome Biol.

[42] Holmes I, Harris K, Quince C (2012). Dirichlet multinomial mixtures: generative models for microbial metagenomics. PLoS One.

[43] Bishop CM, Nasrabadi NM: Pattern recognition and machine learning. Springer; 2006.

[44] Dominique B, Thomas R, Madeleine W, Keir P, Kathy DM, Ian AL. Method for absolute quantification of short chain fatty acids via reverse phase chromatography mass spectrometry. Plos One. 2022;17(4):e0267093. 10.1371/journal.pone.0267093 PMC9020710 35443015

[45] Wang Y, van de Wouw M, Drogos L, Vaghef-Mehrabani E, Reimer RA, Tomfohr-Madsen L, Giesbrecht GF (2022). Sleep and the gut microbiota in preschool-aged children. Sleep.

[46] Clasquin MF, Melamud E, Rabinowitz JD (2012). LC-MS data processing with MAVEN: a metabolomic analysis and visualization engine. Curr Protoc Bioinformatics.

[47] Melamud E, Vastag L, Rabinowitz JD (2010). Metabolomic analysis and visualization engine for LC-MS data. Anal Chem.

[48] Morrison KM, Atkinson SA, Yusuf S, Bourgeois J, McDonald S, McQueen MJ, Persadie R, Hunter B, Pogue J, Teo K (2009). The Family Atherosclerosis Monitoring In earLY life (FAMILY) study: rationale, design, and baseline data of a study examining the early determinants of atherosclerosis. Am Heart J.

[49] Jarman M, Vashi N, Angus A, Bell RC, Giesbrecht GF (2020). Development of a diet quality index to assess adherence to Canadian dietary recommendations in 3-year-old children. Public Health Nutr.

[50] Gasser CE, Kerr JA, Mensah FK, Wake M (2017). Stability and change in dietary scores and patterns across six waves of the Longitudinal Study of Australian Children. Br J Nutr.

[51] Bastiaanssen TFS, Quinn TP, Loughman A (2023). Bugs as features (part 1): concepts and foundations for the compositional data analysis of the microbiome–gut–brain axis. Nature Mental Health.

[52] Pang Z, Zhou G, Ewald J, Chang L, Hacariz O, Basu N, Xia J (2022). Using MetaboAnalyst 5.0 for LC-HRMS spectra processing, multi-omics integration and covariate adjustment of global metabolomics data. Nat Protoc.

[53] Kanehisa M, Goto S (2000). KEGG: kyoto encyclopedia of genes and genomes. Nucleic Acids Res.

[54] Storey JD, Tibshirani R (2003). Statistical significance for genomewide studies. Proc Natl Acad Sci U S A.

[55] Derrien M, Alvarez AS, de Vos WM (2019). The gut microbiota in the first decade of life. Trends Microbiol.

[56] Matsuki T, Yahagi K, Mori H, Matsumoto H, Hara T, Tajima S, Ogawa E, Kodama H, Yamamoto K, Yamada T (2016). A key genetic factor for fucosyllactose utilization affects infant gut microbiota development. Nat Commun.

[57] Roswall J, Olsson LM, Kovatcheva-Datchary P, Nilsson S, Tremaroli V, Simon MC, Kiilerich P, Akrami R, Kramer M, Uhlen M (2021). Developmental trajectory of the healthy human gut microbiota during the first 5 years of life. Cell Host Microbe.

[58] Beller L, Deboutte W, Falony G, Vieira-Silva S, Tito RY, Valles-Colomer M, Rymenans L, Jansen D, Van Espen L, Papadaki MI (2021). Successional stages in infant gut microbiota maturation. mBio.

[59] Stewart CJ, Ajami NJ, O'Brien JL, Hutchinson DS, Smith DP, Wong MC, Ross MC, Lloyd RE, Doddapaneni H, Metcalf GA (2018). Temporal development of the gut microbiome in early childhood from the TEDDY study. Nature.

[60] Wang Y, Chen X, Yu Y, Liu Y, Zhang Q, Bai J (2020). Association between gut microbiota and infant's temperament in the first year of life in a Chinese birth cohort. Microorganisms.

[61] Kelsey CM, Prescott S, McCulloch JA, Trinchieri G, Valladares TL, Dreisbach C, Alhusen J, Grossmann T (2021). Gut microbiota composition is associated with newborn functional brain connectivity and behavioral temperament. Brain Behav Immun.

[62] Fox M, Lee SM, Wiley KS, Lagishetty V, Sandman CA, Jacobs JP, Glynn LM: Development of the infant gut microbiome predicts temperament across the first year of life. Dev Psychopathol 2021:1-12. https://pubmed.ncbi.nlm.nih.gov/34108055/. 10.1017/S0954579421000456 PMC9463039 34108055

[63] Guzzardi MA, Ederveen THA, Rizzo F, Weisz A, Collado MC, Muratori F, Gross G, Alkema W, Iozzo P (2022). Maternal pre-pregnancy overweight and neonatal gut bacterial colonization are associated with cognitive development and gut microbiota composition in pre-school-age offspring. Brain Behav Immun.

[64] Tamana SK, Tun HM, Konya T, Chari RS, Field CJ, Guttman DS, Becker AB, Moraes TJ, Turvey SE, Subbarao P (2021). Bacteroides-dominant gut microbiome of late infancy is associated with enhanced neurodevelopment. Gut Microbes.

[65] Matsumoto M, Ooga T, Kibe R, Aiba Y, Koga Y, Benno Y (2017). Colonic absorption of low-molecular-weight metabolites influenced by the intestinal microbiome: a pilot study. PLoS One.

[66] Brosnan ME, Brosnan JT (2020). Histidine metabolism and function. J Nutr.

[67] Panula P, Sundvik M, Karlstedt K (2014). Developmental roles of brain histamine. Trends Neurosci.

[68] Costea PI, Hildebrand F, Arumugam M, Backhed F, Blaser MJ, Bushman FD, de Vos WM, Ehrlich SD, Fraser CM, Hattori M (2018). Enterotypes in the landscape of gut microbial community composition. Nat Microbiol.

[69] Vieira-Silva S, Falony G, Darzi Y, Lima-Mendez G, Garcia Yunta R, Okuda S, Vandeputte D, Valles-Colomer M, Hildebrand F, Chaffron S, Raes J (2016). Species-function relationships shape ecological properties of the human gut microbiome. Nat Microbiol.

[70] Knights D, Ward TL, McKinlay CE, Miller H, Gonzalez A, McDonald D, Knight R (2014). Rethinking "enterotypes". Cell Host Microbe.

[71] Cheng M, Ning K (2019). Stereotypes about enterotype: the old and new ideas. Genomics Proteomics Bioinformatics.

[72] Xiao L, Wang J, Zheng J, Li X, Zhao F (2021). Deterministic transition of enterotypes shapes the infant gut microbiome at an early age. Genome Biol.

[73] Carlson AL, Xia K, Azcarate-Peril MA, Goldman BD, Ahn M, Styner MA, Thompson AL, Geng X, Gilmore JH, Knickmeyer RC (2018). Infant gut microbiome associated with cognitive development. Biol Psychiatry.

[74] Narusyte J, Ropponen A, Alexanderson K, Svedberg P (2017). Internalizing and externalizing problems in childhood and adolescence as predictors of work incapacity in young adulthood. Soc Psychiatry Psychiatr Epidemiol.

[75] Roager HM, Stanton C, Hall LJ (2023). Microbial metabolites as modulators of the infant gut microbiome and host-microbial interactions in early life. Gut Microbes.

[76] Klimenko NS, Odintsova VE, Revel-Muroz A, Tyakht AV (2022). The hallmarks of dietary intervention-resilient gut microbiome. NPJ Biofilms Microbiomes.

[77] Hjorth MF, Blaedel T, Bendtsen LQ, Lorenzen JK, Holm JB, Kiilerich P, Roager HM, Kristiansen K, Larsen LH, Astrup A (2019). Prevotella-to-Bacteroides ratio predicts body weight and fat loss success on 24-week diets varying in macronutrient composition and dietary fiber: results from a post-hoc analysis. Int J Obes (Lond).

[78] Hjorth MF, Roager HM, Larsen TM, Poulsen SK, Licht TR, Bahl MI, Zohar Y, Astrup A (2018). Pre-treatment microbial Prevotella-to-Bacteroides ratio, determines body fat loss success during a 6-month randomized controlled diet intervention. Int J Obes (Lond).

[79] Christensen L, Roager HM, Astrup A, Hjorth MF (2018). Microbial enterotypes in personalized nutrition and obesity management. Am J Clin Nutr.

[80] Gu Y, Wang X, Li J, Zhang Y, Zhong H, Liu R, Zhang D, Feng Q, Xie X, Hong J (2017). Analyses of gut microbiota and plasma bile acids enable stratification of patients for antidiabetic treatment. Nat Commun.

