# Supplementary material for: Cluster-specific associations between the gut microbiota and behavioral outcomes in preschool-aged children
Source: Microbiome. 2024 Mar 21;12:60. doi: 10.1186/s40168-024-01773-5 (PMC10956200; doi:10.1186/s40168-024-01773-5)
Supplement: Supplementary file 2 — Additional file 1: Figure S1. Gut microbiota clustering using Dirichlet multinomial mixtures. Figure S2. Flowchart of data analysis approach. Figure S3. Differences in stool metabolic pathways between the four clusters. Figure S4. Differences in stool nucleotide levels between the four clusters. Figure S5. Behavioral Symptom scores between the four clusters. Figure S6. Number of microbial taxa that correlate with stool histidine to urocanate turnover. Figure S7. Relationship between Social Skills and urocanate metabolism for participants with low histidine-urocanate turnover rates. Figure S8. Levels of stool urocanate and histidine-urocanate turnover between children with “at risk” and “not at risk” scores for Social Skills and Developmental Social Disorders. Table S1. Sample characteristics of all combined participants. Table S2. PERMANOVA results for stool metabolome beta diversity. Individual independent effects are depicted. Table S3. ANCOVA Results for Adaptive Skill Scores While Correction for Gestational Age at Birth, Birth Weight, Ethnicity and Child Grain Intake. Table S4. ANCOVA Results for Developmental Social Disorder Scores While Correction for Gestational Age at Birth, Birth Weight, Ethnicity and Child Grain Intake. Table S5. ANCOVA Results for Social Skill Scores While Correction for Gestational Age at Birth, Birth Weight, Ethnicity and Child Grain Intake. Table S6. ANCOVA Results for Functional Communication Scores While Correction for Gestational Age at Birth, Birth Weight, Ethnicity and Child Grain Intake. Table S7. ANCOVA Results for Daily Living Scores While Correction for Gestational Age at Birth, Birth Weight, Ethnicity and Child Grain Intake. [file 40168_2024_1773_MOESM1_ESM.docx]

**Supplementary Data**

**Cluster-Specific Associations Between the Gut Microbiota and Behavioral Outcomes in Preschool-Aged Children**

Marcel van de Wouw^1^, Yanan Wang^1,8^, **Matthew L. Workentine^5^**, Elnaz Vaghef-Mehrabani^1,3^, Delaney Barth^1^, Emily M. Mercer^1, 9, 10^, Deborah Dewey^1,3,4,11^, Marie-Claire Arrieta^1, 9, 10^, Raylene A. Reimer^3,6,7^, Lianne Tomfohr-Madsen^1,2,3,12^, Gerald F. Giesbrecht^1,2,3,4^

1. Department of Pediatrics, University of Calgary, Calgary, Alberta, Canada
2. Department of Psychology, University of Calgary, Calgary, Alberta, Canada
3. Alberta Children’s Hospital Research Institute (ACHRI), University of Calgary, Calgary, Alberta, Canada
4. Department of Community Health Sciences, University of Calgary, Calgary, Alberta, Canada
5. UCVM Bioinformatics, Faculty of Veterinary Medicine, University of Calgary, Calgary, Alberta, Canada
6. Faculty of Kinesiology, University of Calgary, Calgary, Alberta, Canada
7. Department of Biochemistry and Molecular Biology, Cumming School of Medicine, University of Calgary, Alberta, Canada
8. Microbiomes for One Systems Health, Health & Biosecurity, CSIRO, Adelaide, SA, Australia
9. Department of Physiology and Pharmacology, University of Calgary, Calgary, AB, Canada.
10. International Microbiome Centre, University of Calgary, Calgary, Alberta, Canada
11. Hotchkiss Brain Institute (HBI), University of Calgary, Calgary, Alberta, Canada
12. Faculty of Education, University of British Columbia, Vancouver, British Columbia, Canada

**Address correspondence to**: Gerald F. Giesbrecht, PhD, Department of Psychology, University of Calgary, 2500 University Drive, NW, Calgary, AB, T2N 1N4, ggiesbre@ucalgary.ca.

### Figure S1. Gut microbiota clustering using Dirichlet multinomial mixtures


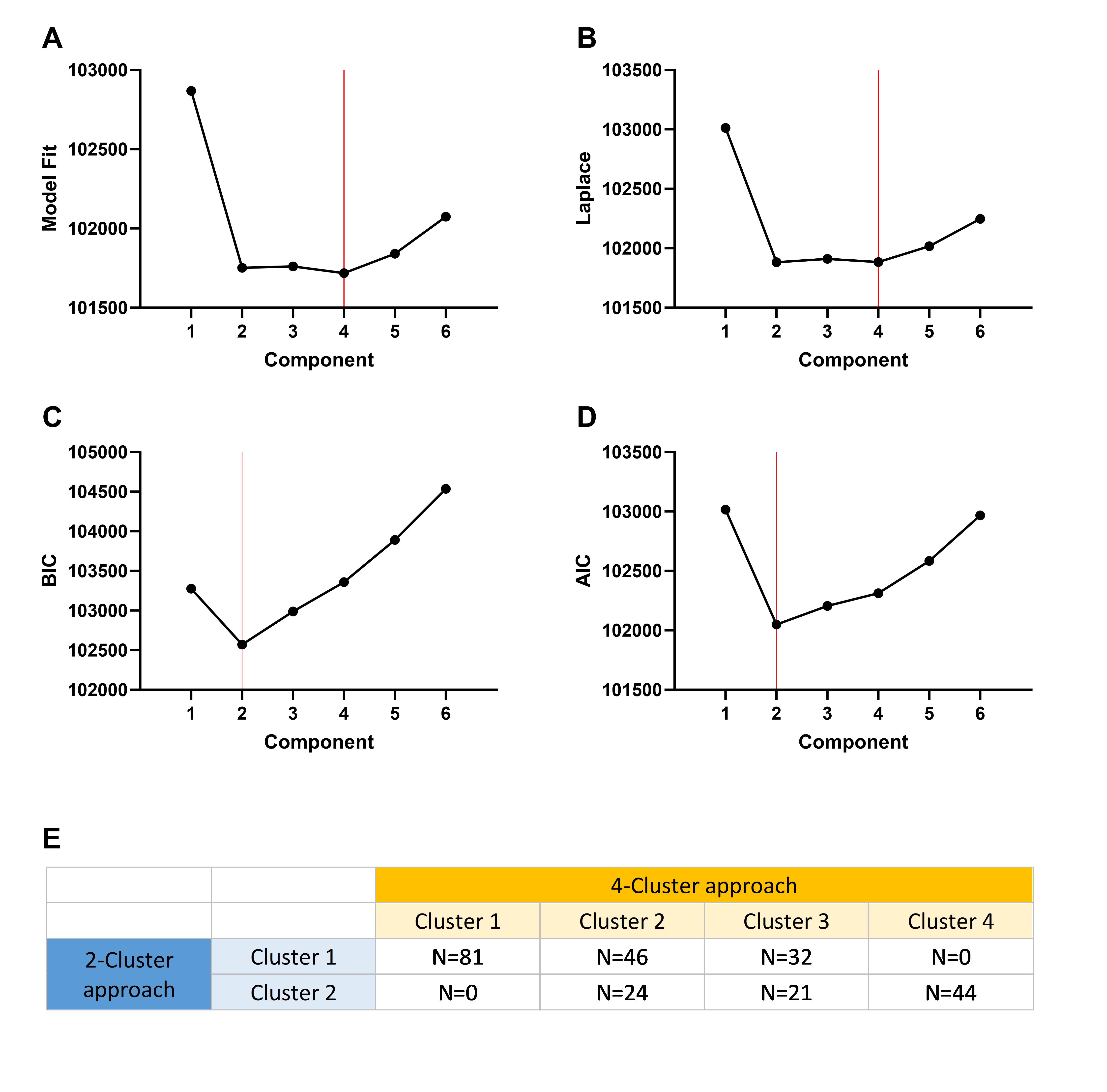


Measures for selecting the optimal number of gut microbiome clusters using Dirichlet multinomial mixtures (DMM) are depicted for Model Fit (A), Laplace (B), Bayesian Information Criterion (BIC) (C), and Aikaike Information Criterion (AIC). The optimal number of clusters was determined using the Laplace approximation. BIC and ACI indicate that a 2-cluster approach is more optimal, so participant allocation based on clustering approaches was assessed (E), which showed considerable overlap between both approaches, suggesting that the 4-cluster approach encompasses the 2-cluster approach while being more nuanced.

### Figure S2. Flowchart of data analysis approach


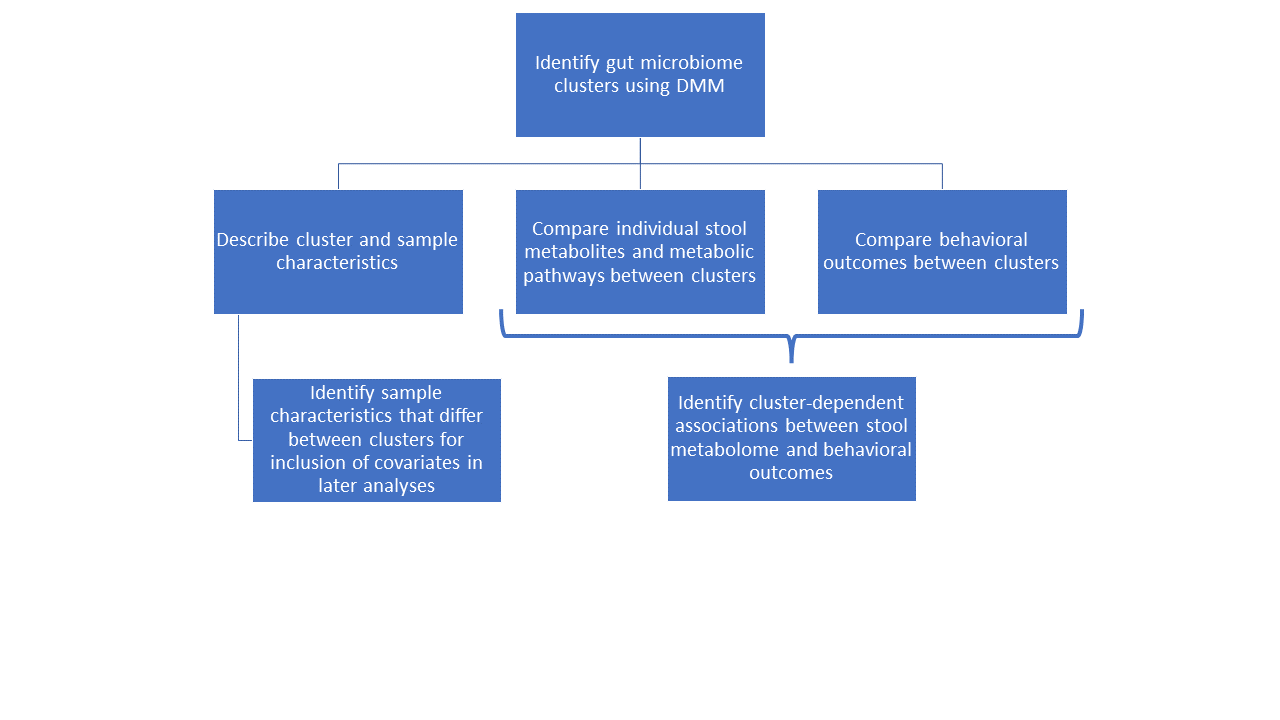


Participant gut microbiota were clustered using Dirichlet multinomial mixtures (DMM), after which cluster and sample characteristics were described and covariates for follow-up analyses were identified. Differences in individual stool metabolites and metabolic pathways (identified using Metaboanalyst) were subsequently investigated, as well as behavioral differences between clusters. Finally, gut microbiota-dependent associations between the stool metabolome and behavioral outcomes were investigated.

### Figure S3. Differences in stool metabolic pathways between the four clusters


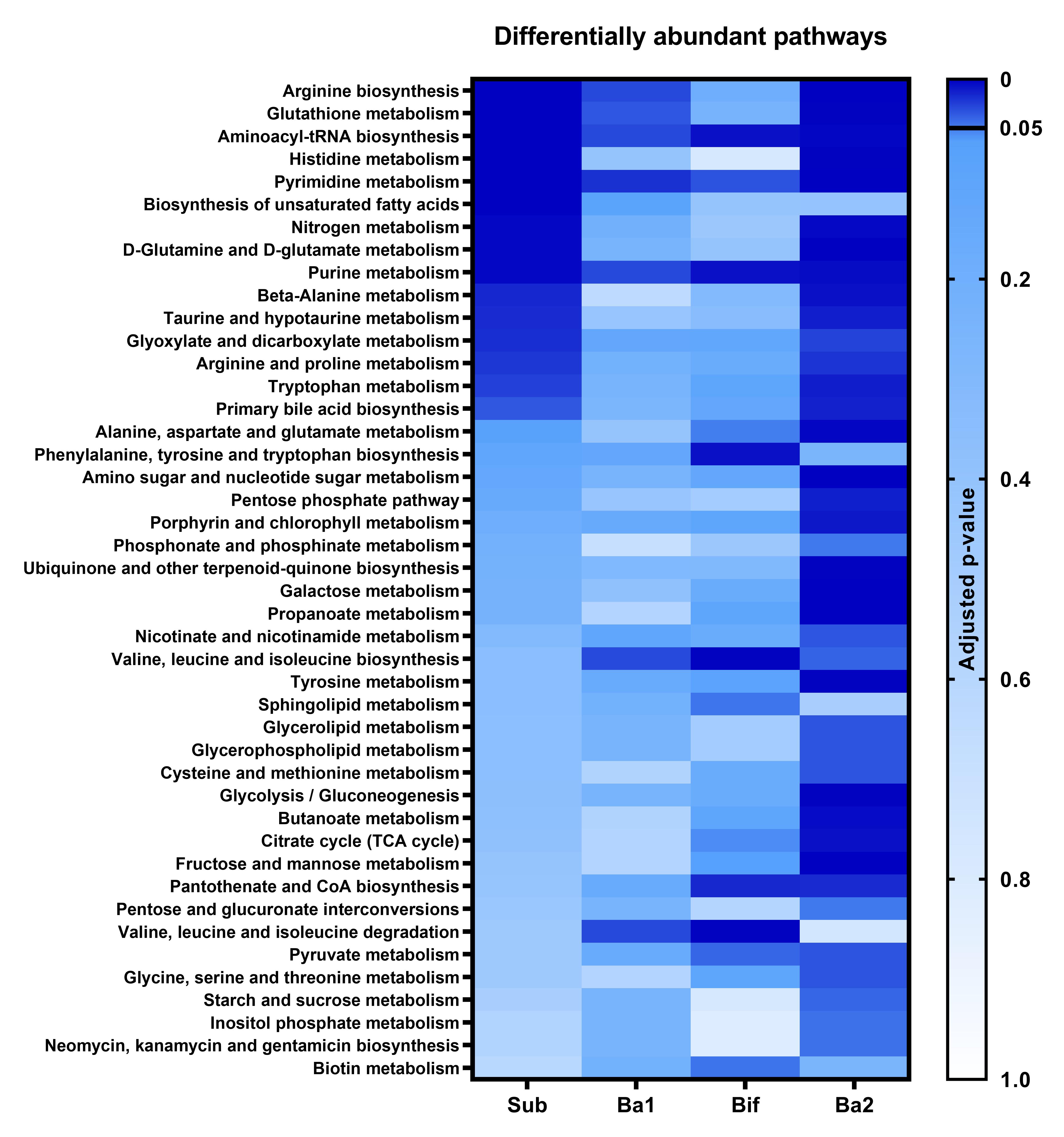


Differentially abundant pathways were subsequently compared between the clusters using Metaboanalyst and the statistically significant different ones were also depicted in a heatmap. Total n=248, cluster sub is n=81, Ba1 is n=70, Bif is n=53, Ba2 is n=44.

### Figure S4. Differences in stool nucleotide levels between the four clusters


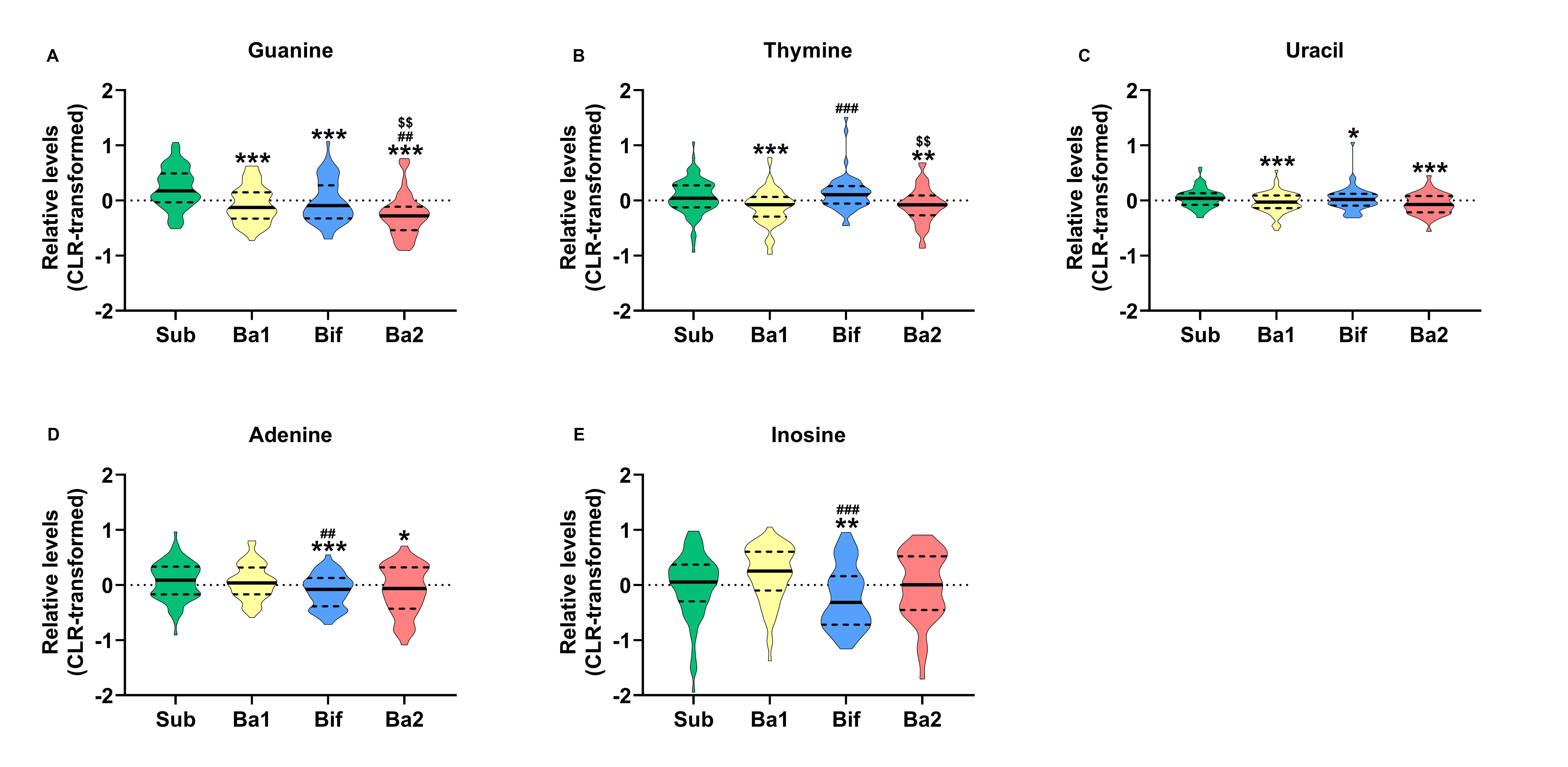


Relative nucleotide levels were quantified from stool samples (A-E). There were overall differences between the four clusters for guanine (χ2(3) = 44.423, *p* < 0.001), thymine (χ2(3) = 21.410, *p* < 0.001), uracil (χ2(3) = 16.290, *p* < 0.001), adenine (χ2(3) = 14.205, *p* < 0.001), and inosine (χ2(3) = 16.837, *p* < 0.001). Statistical differences were assessed using Kruskal-Wallis tests followed by Mann-Whitney tests. Data are depicted as violin plots where the middle line is the median and the dotted lines are quartiles. The grey zones depict the scores at which a participant would be “at risk”, for an associated disorder. Total n=248, cluster sub is n=81, Ba1 is n=70, Bif is n=53, Ba2 is n=44. For the statistical significance, *p<0.05; **p<0.01; ***p<0.001, * indicates a statistical difference compared to the Sub cluster, ^#^ compared to the Ba1 cluster, ^$^ compared to the Bif cluster.

### Figure S5. Behavioral Symptom scores between the four clusters


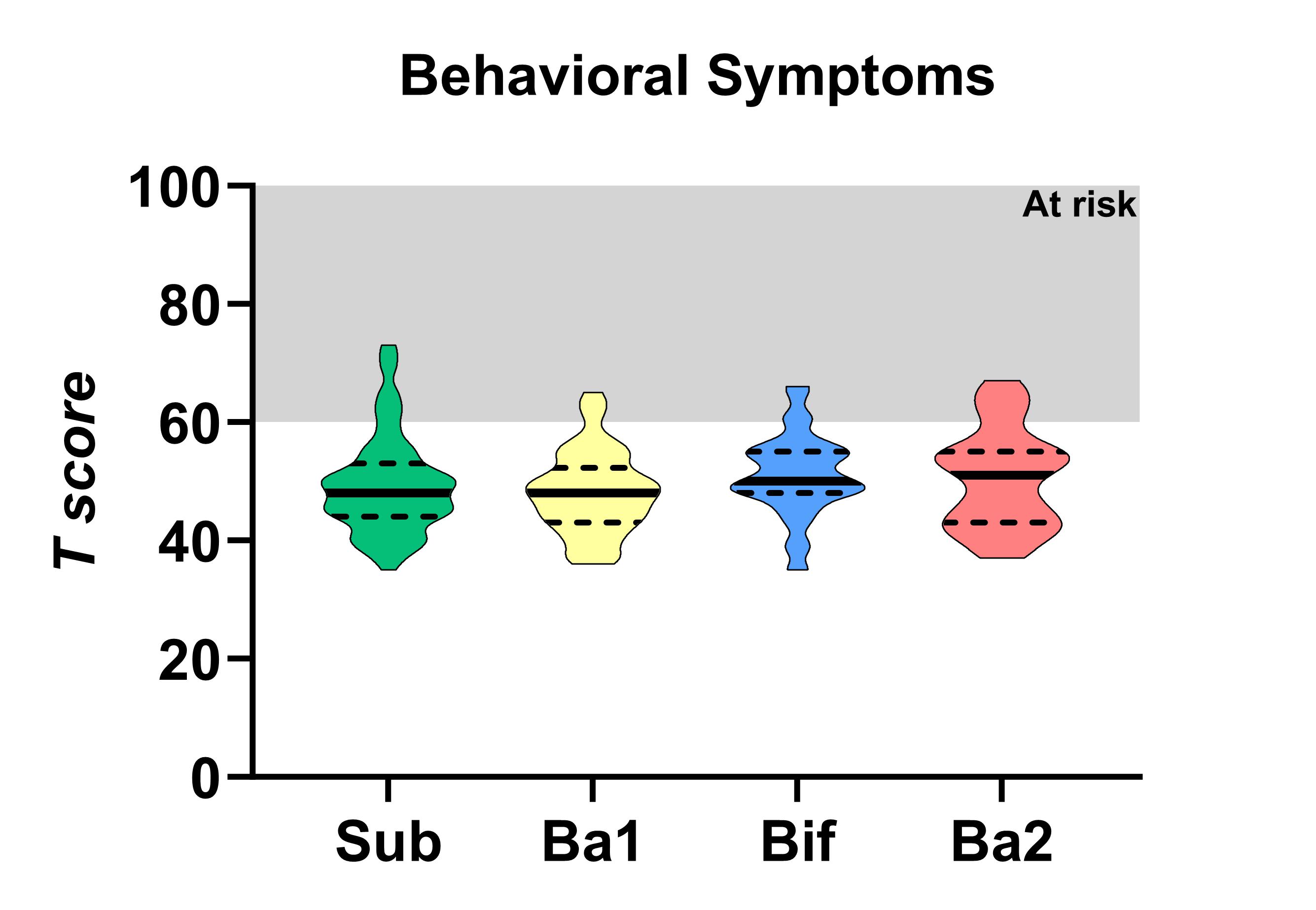


The BASC-2 was used to assessed differences in Behavioral Symptoms between the clusters. Data are depicted as violin plots where the middle line is the median and the dotted lines are quartiles. The grey zones depict the scores at which a participant would be “at risk”, for an associated disorder. Total n=248, cluster sub is n=81, Ba1 is n=70, Bif is n=53, Ba2 is n=44.

### Figure S6. Number of microbial taxa that correlate with stool histidine to urocanate turnover


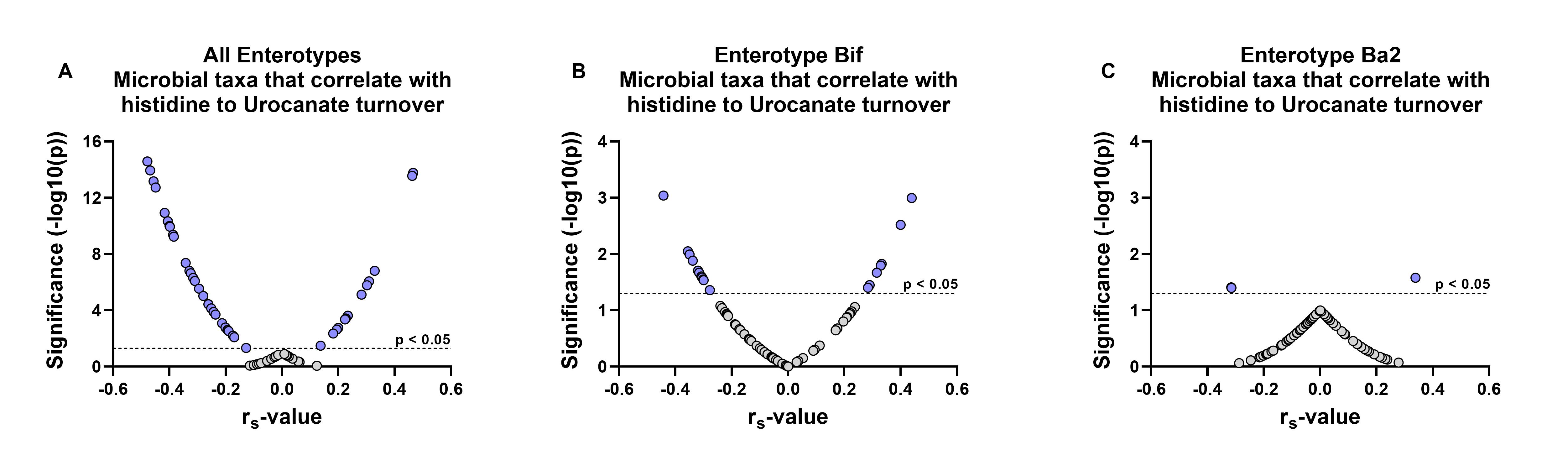


To investigate the differential involvement of the gut microbiota in stool histidine to urocanate turnover between clusters, all bacterial taxa were correlated using data from all participants (A), only those that were in the Bif cluster (B), or those in the Ba2 cluster (C). Of the investigated 69 taxa, 46 correlated with histidine to urocanate turnover in the entire population (min r_s_ = -0.489, max r_s_ = 0.466), 19 when only investigating the Bif cluster participants (min r_s_ = -0.442, max r_s_ = 0.439), and 3 when only investigating the Ba2 cluster participants (min r_s_ = -0.316, max r_s_ = 0.339). Total n=248, Bif is n=53, Ba2 is n=44.

### Figure S7. Relationship between Social Skills and urocanate metabolism for participants with low histidine-urocanate turnover rates


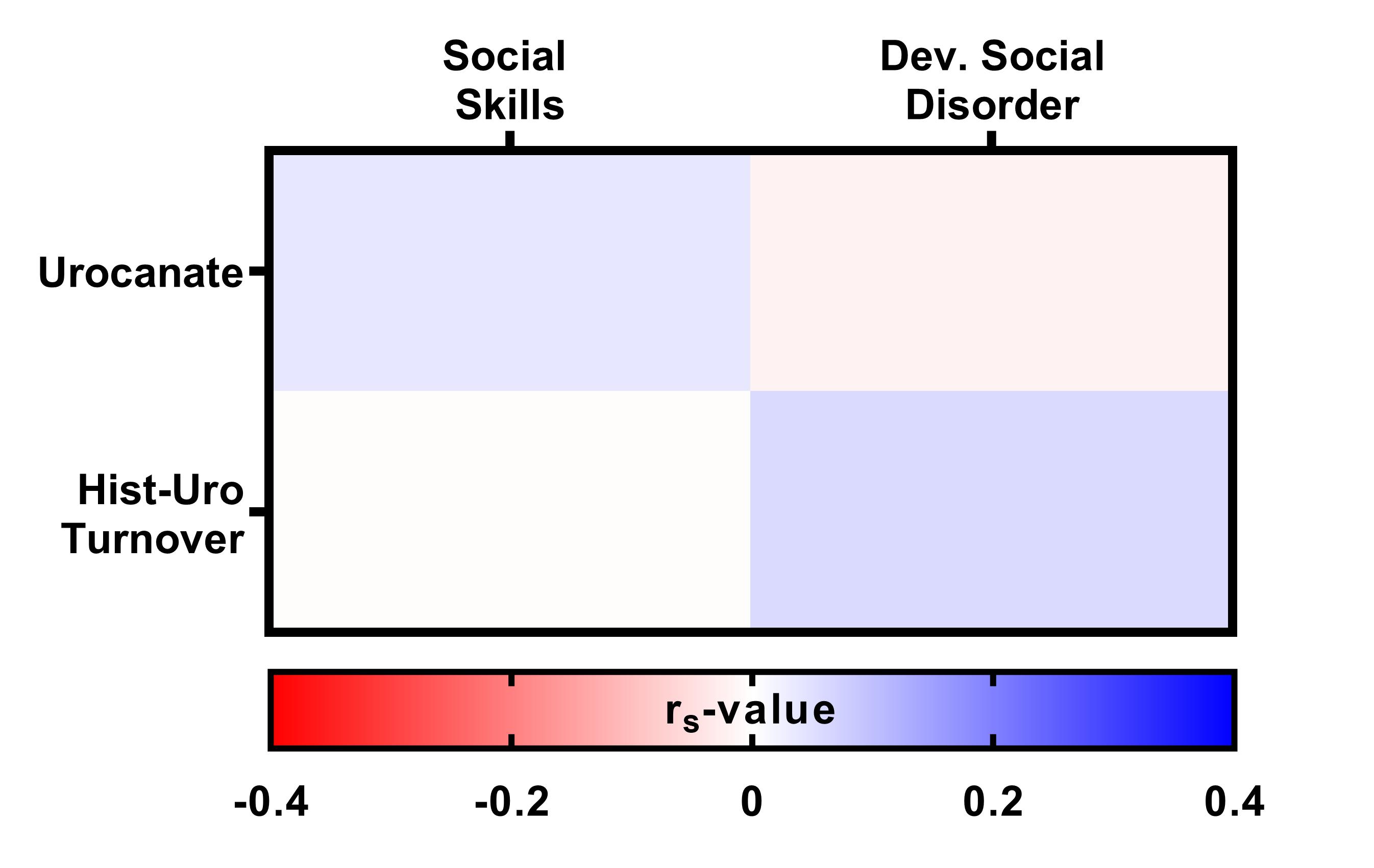


After selecting all participants (n = 64) with a histidine-urocanate turnover rate >2 (the median turnover rate for cluster Ba2 was 1.75), there was no correlation between Social Skills and urocanate (r_s_ = 0.037, *p* = 0.772), as well as histidine-urocanate turnover rates (r_s_ = -0.005, *p* = 0.969). In addition, no correlation was observed between Developmental Social Disorders scores and urocanate (r_s_ = -0.021, *p* = 0.867), as well as histidine-urocanate turnover rates (r_s_ = 0.057, *p* = 0.655).

### Figure S8. Levels of stool urocanate and histidine-urocanate turnover between children with “at risk” and “not at risk” scores for Social Skills and Developmental Social Disorders


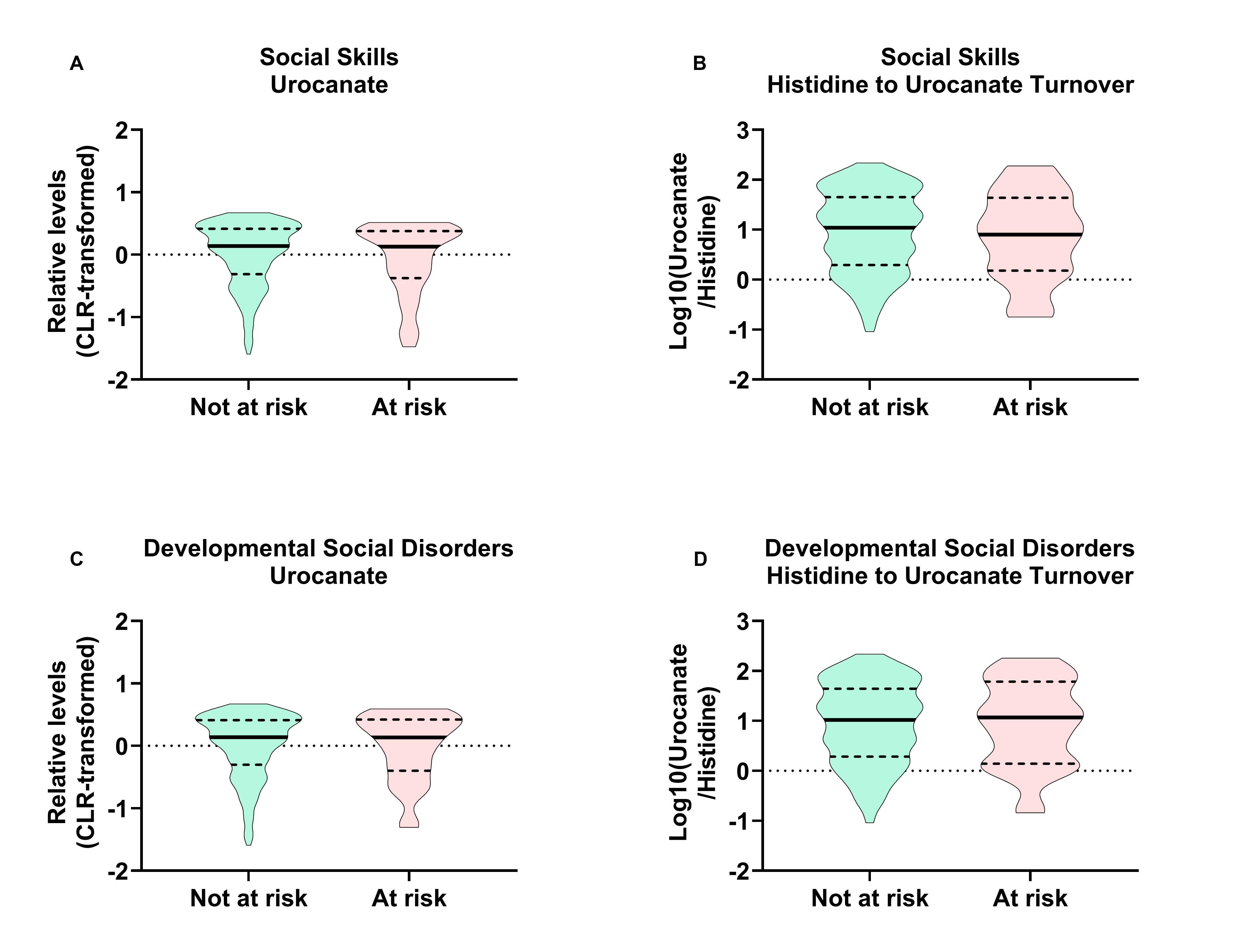


No differences were found in stool levels of urocanate and histidine-urocanate turnover between children with “at risk” and “not at risk” scores for Social Skills and Developmental Social Disorders (A-D). The cut-off scores for being at risk for Social Skills is any score lower then 40 (including 40) (n=31), and for Developmental Social Disorders any score above 60 (including 60) (n=29). Mann-Whitney U tests between the groups were as follows: A) Urocanate vs Social Skills (U = 3007.0, *p* = 0.523), B) histidine-urocanate turnover vs Social Skills (U = 3031.0, *p* = 0.567), C) Urocanate vs Developmental Social Disorders (U = 3058.0, *p* = 0.806), D) histidine-urocanate turnover vs Developmental Social Disorders (U = 3144.0, *p* = 0.994).

### Table S1. Sample characteristics of all combined participants

| **Maternal characteristics** |  |
| --- | --- |
| Age (years), Mean (SD, range) | 32.4 (4.1, 20.3-44.4) |
| Education, N (%) |  |
| *Completed high school diploma* | 14 (5.6%) |
| *Completed trade, technical diploma* | 46 (18.5%) |
| *Completed university degree* | 137 (55.2%) |
| *Completed post-graduate degree* | 51 (20.6%) |
| Annual household income, N (%)^b^ |  |
| *<$20,000* | 5 (2.0%) |
| *$20, 000 – $39,999* | 8 (3.2%) |
| *$40,000 – $69,9999* | 27 (10.9%) |
| *$70,000 – $99,9999* | 57 (23.1%) |
| *>$100,000* | 150 (60.7%) |
| Ethnicity, N (%) |  |
| *White* | 214 (86.3%) |
| *Chinese* | 10 (4.0%) |
| *South-East Asian* | 7 (2.8%) |
| *Latin America* | 6 (2.4%) |
| *Other* | 11 (4.4%) |
| **Child characteristics** |  |
| Age (years), Mean (SD, range) | 4.37 (0.48, 3.05-5.00) |
| Male, N (%) | 130 (52.4%) |
| Mode of delivery – Vaginal, N (%)^b^ | 187 (75.4%) |
| Gestational age (weeks), Mean (SD, range)^b^ | 39.1 (1.8, 29.4-42.0) |
| Birth weight (gram), Mean (SD, range)^b^ | 3334 (585, 1200-5210) |
| Antibiotic exposure – Yes, N (%)^c^ | 97 (39.1%) |
| BASC-2, Mean (SD, range)^b^ |  |
| *Adaptive Skills (T score)* | 50.8 (8.3, 24-68) |
| *Internalizing (T score)* | 48.1 (8.0, 30-67) |
| *Externalizing (T score)* | 49.3 (8.0, 34-74) |
| *Behavioural Symptoms (T score)* | 49.6 (7.7, 35-73) |
| Diet, Mean (SD, range)^d^ |  |
| *Diet quality score* | 3.70 (0.67, 1.95-5.74) |
| *Total energy intake (calories)* | 1567 (422, 614-2833) |
| *Vegetable and fruit (gram)* | 480 (200, 92-1304) |
| *Grain product (gram)* | 144 (72, 12.7-483) |
| *Meat and alternatives (gram)* | 110 (50, 11-370) |

### Table S2. PERMANOVA results for stool metabolome beta diversity. Individual independent effects are depicted.

|  | Df | SumOfSqs | R2 | F | Pr(>F) |
| --- | --- | --- | --- | --- | --- |
| Child Sex | 1 | 152.7103 | 0.006604 | 1.298971 | 0.165834 |
| Child Age (Years) | 1 | 129.2794 | 0.005591 | 1.099665 | 0.304695 |
| Ethnicity | 1 | 249.6159 | 0.010795 | 2.12326 | 0.005994 |
| Socioeconomic status | 1 | 113.9172 | 0.004927 | 0.968992 | 0.4995 |
| Gestational age at birth | 1 | 143.5535 | 0.006208 | 1.221082 | 0.195804 |
| Delivery mode | 1 | 136.3749 | 0.005898 | 1.16002 | 0.254745 |
| Antibiotic exposure | 1 | 92.20687 | 0.003988 | 0.784322 | 0.77023 |
| Diet quality score | 1 | 243.2777 | 0.010521 | 2.069347 | 0.006993 |
| Grain product consumption | 1 | 112.3582 | 0.004859 | 0.955731 | 0.486513 |
| Residual | 185 | 21749.08 | 0.940608 |  |  |
| Total | 194 | 23122.37 | 1 |  |  |

### Table S3. ANCOVA Results for Adaptive Skill Scores While Correction for Gestational Age at Birth, Birth Weight, Ethnicity and Child Grain Intake.

|  |  | **Unadjusted model** | | | | **Adjusted model** | | | |
| --- | --- | --- | --- | --- | --- | --- | --- | --- | --- |
| (I) Cluster | (J) Cluster | Mean Difference (I-J) | Std. Error | df | Sig. | Mean Difference (I-J) | Std. Error | df | Sig. |
| Sub | Ba1 | -2.288 | 1.340 | 243.000 | 0.089 | -1.828 | 1.434 | 185.000 | 0.204 |
|  | Bif | 2.637 | 1.459 | 243.000 | 0.072 | 3.149 | 1.738 | 185.000 | 0.072 |
|  | Ba2 | 1.244 | 1.537 | 243.000 | 0.419 | 1.352 | 1.856 | 185.000 | 0.467 |
| Ba1 | Sub | 2.288 | 1.340 | 243.000 | 0.089 | 1.828 | 1.434 | 185.000 | 0.204 |
|  | Bif | 4.926 | 1.503 | 243.000 | 0.001 | 4.977 | 1.749 | 185.000 | 0.005 |
|  | Ba2 | 3.532 | 1.579 | 243.000 | 0.026 | 3.181 | 1.834 | 185.000 | 0.085 |
| Bif | Sub | -2.637 | 1.459 | 243.000 | 0.072 | -3.149 | 1.738 | 185.000 | 0.072 |
|  | Ba1 | -4.926 | 1.503 | 243.000 | 0.001 | -4.977 | 1.749 | 185.000 | 0.005 |
|  | Ba2 | -1.393 | 1.681 | 243.000 | 0.408 | -1.797 | 2.030 | 185.000 | 0.377 |
| Ba2 | Sub | -1.244 | 1.537 | 243.000 | 0.419 | -1.352 | 1.856 | 185.000 | 0.467 |
|  | Ba1 | -3.532 | 1.579 | 243.000 | 0.026 | -3.181 | 1.834 | 185.000 | 0.085 |
|  | Bif | 1.393 | 1.681 | 243.000 | 0.408 | 1.797 | 2.030 | 185.000 | 0.377 |

### Table S4. ANCOVA Results for Developmental Social Disorder Scores While Correction for Gestational Age at Birth, Birth Weight, Ethnicity and Child Grain Intake.

|  |  | **Unadjusted model** | | | | **Adjusted model** | | | |
| --- | --- | --- | --- | --- | --- | --- | --- | --- | --- |
| (I) Cluster | (J) Cluster | Mean Difference (I-J) | Std. Error | df | Sig. | Mean Difference (I-J) | Std. Error | df | Sig. |
| Sub | Ba1 | 0.395 | 1.287 | 243.000 | 0.759 | 0.527 | 1.351 | 185.000 | 0.697 |
|  | Bif | -3.004 | 1.401 | 243.000 | 0.033 | -1.868 | 1.638 | 185.000 | 0.256 |
|  | Ba2 | -2.494 | 1.477 | 243.000 | 0.093 | -2.154 | 1.749 | 185.000 | 0.220 |
| Ba1 | Sub | -0.395 | 1.287 | 243.000 | 0.759 | -0.527 | 1.351 | 185.000 | 0.697 |
|  | Bif | -3.399 | 1.444 | 243.000 | 0.019 | -2.395 | 1.648 | 185.000 | 0.148 |
|  | Ba2 | -2.889 | 1.517 | 243.000 | 0.058 | -2.680 | 1.728 | 185.000 | 0.123 |
| Bif | Sub | 3.004 | 1.401 | 243.000 | 0.033 | 1.868 | 1.638 | 185.000 | 0.256 |
|  | Ba1 | 3.399 | 1.444 | 243.000 | 0.019 | 2.395 | 1.648 | 185.000 | 0.148 |
|  | Ba2 | 0.510 | 1.615 | 243.000 | 0.752 | -0.286 | 1.913 | 185.000 | 0.881 |
| Ba2 | Sub | 2.494 | 1.477 | 243.000 | 0.093 | 2.154 | 1.749 | 185.000 | 0.220 |
|  | Ba1 | 2.889 | 1.517 | 243.000 | 0.058 | 2.680 | 1.728 | 185.000 | 0.123 |
|  | Bif | -0.510 | 1.615 | 243.000 | 0.752 | 0.286 | 1.913 | 185.000 | 0.881 |

### Table S5. ANCOVA Results for Social Skill Scores While Correction for Gestational Age at Birth, Birth Weight, Ethnicity and Child Grain Intake.

|  |  | **Unadjusted model** | | | | **Adjusted model** | | | |
| --- | --- | --- | --- | --- | --- | --- | --- | --- | --- |
| (I) Cluster | (J) Cluster | Mean Difference (I-J) | Std. Error | df | Sig. | Mean Difference (I-J) | Std. Error | df | Sig. |
| Sub | Ba1 | -2.269 | 1.422 | 243.000 | 0.112 | -1.902 | 1.526 | 185.000 | 0.214 |
|  | Bif | 1.894 | 1.549 | 243.000 | 0.223 | 2.436 | 1.849 | 185.000 | 0.189 |
|  | Ba2 | 2.418 | 1.632 | 243.000 | 0.140 | 2.062 | 1.975 | 185.000 | 0.298 |
| Ba1 | Sub | 2.269 | 1.422 | 243.000 | 0.112 | 1.902 | 1.526 | 185.000 | 0.214 |
|  | Bif | 4.163 | 1.595 | 243.000 | 0.010 | 4.338 | 1.861 | 185.000 | 0.021 |
|  | Ba2 | 4.688 | 1.677 | 243.000 | 0.006 | 3.965 | 1.952 | 185.000 | 0.044 |
| Bif | Sub | -1.894 | 1.549 | 243.000 | 0.223 | -2.436 | 1.849 | 185.000 | 0.189 |
|  | Ba1 | -4.163 | 1.595 | 243.000 | 0.010 | -4.338 | 1.861 | 185.000 | 0.021 |
|  | Ba2 | 0.524 | 1.785 | 243.000 | 0.769 | -0.374 | 2.161 | 185.000 | 0.863 |
| Ba2 | Sub | -2.418 | 1.632 | 243.000 | 0.140 | -2.062 | 1.975 | 185.000 | 0.298 |
|  | Ba1 | -4.688 | 1.677 | 243.000 | 0.006 | -3.965 | 1.952 | 185.000 | 0.044 |
|  | Bif | -0.524 | 1.785 | 243.000 | 0.769 | 0.374 | 2.161 | 185.000 | 0.863 |

### Table S6. ANCOVA Results for Functional Communication Scores While Correction for Gestational Age at Birth, Birth Weight, Ethnicity and Child Grain Intake.

|  |  | **Unadjusted model** | | | | **Adjusted model** | | | |
| --- | --- | --- | --- | --- | --- | --- | --- | --- | --- |
| (I) Cluster | (J) Cluster | Mean Difference (I-J) | Std. Error | df | Sig. | Mean Difference (I-J) | Std. Error | df | Sig. |
| Sub | Ba1 | -2.841 | 1.223 | 243.000 | 0.021 | -2.935 | 1.352 | 185.000 | 0.031 |
|  | Bif | 2.375 | 1.332 | 243.000 | 0.076 | 1.471 | 1.640 | 185.000 | 0.371 |
|  | Ba2 | -0.945 | 1.404 | 243.000 | 0.501 | -1.022 | 1.751 | 185.000 | 0.560 |
| Ba1 | Sub | 2.841 | 1.223 | 243.000 | 0.021 | 2.935 | 1.352 | 185.000 | 0.031 |
|  | Bif | 5.215 | 1.372 | 243.000 | 0.000 | 4.406 | 1.650 | 185.000 | 0.008 |
|  | Ba2 | 1.895 | 1.442 | 243.000 | 0.190 | 1.913 | 1.730 | 185.000 | 0.270 |
| Bif | Sub | -2.375 | 1.332 | 243.000 | 0.076 | -1.471 | 1.640 | 185.000 | 0.371 |
|  | Ba1 | -5.215 | 1.372 | 243.000 | 0.000 | -4.406 | 1.650 | 185.000 | 0.008 |
|  | Ba2 | -3.320 | 1.535 | 243.000 | 0.032 | -2.493 | 1.915 | 185.000 | 0.195 |
| Ba2 | Sub | 0.945 | 1.404 | 243.000 | 0.501 | 1.022 | 1.751 | 185.000 | 0.560 |
|  | Ba1 | -1.895 | 1.442 | 243.000 | 0.190 | -1.913 | 1.730 | 185.000 | 0.270 |
|  | Bif | 3.320 | 1.535 | 243.000 | 0.032 | 2.493 | 1.915 | 185.000 | 0.195 |

### Table S7. ANCOVA Results for Daily Living Scores While Correction for Gestational Age at Birth, Birth Weight, Ethnicity and Child Grain Intake.

|  |  | **Unadjusted model** | | | | **Adjusted model** | | | |
| --- | --- | --- | --- | --- | --- | --- | --- | --- | --- |
| (I) Cluster | (J) Cluster | Mean Difference (I-J) | Std. Error | df | Sig. | Mean Difference (I-J) | Std. Error | df | Sig. |
| Sub | Ba1 | -2.457 | 1.496 | 243.000 | 0.102 | -1.272 | 1.544 | 186.000 | 0.411 |
|  | Bif | 2.159 | 1.629 | 243.000 | 0.186 | 4.470 | 1.872 | 186.000 | 0.018 |
|  | Ba2 | 0.793 | 1.717 | 243.000 | 0.644 | 2.077 | 1.999 | 186.000 | 0.300 |
| Ba1 | Sub | 2.457 | 1.496 | 243.000 | 0.102 | 1.272 | 1.544 | 186.000 | 0.411 |
|  | Bif | 4.615 | 1.678 | 243.000 | 0.006 | 5.742 | 1.884 | 186.000 | 0.003 |
|  | Ba2 | 3.250 | 1.763 | 243.000 | 0.067 | 3.349 | 1.975 | 186.000 | 0.092 |
| Bif | Sub | -2.159 | 1.629 | 243.000 | 0.186 | -4.470 | 1.872 | 186.000 | 0.018 |
|  | Ba1 | -4.615 | 1.678 | 243.000 | 0.006 | -5.742 | 1.884 | 186.000 | 0.003 |
|  | Ba2 | -1.365 | 1.878 | 243.000 | 0.468 | -2.393 | 2.187 | 186.000 | 0.275 |
| Ba2 | Sub | -0.793 | 1.717 | 243.000 | 0.644 | -2.077 | 1.999 | 186.000 | 0.300 |
|  | Ba1 | -3.250 | 1.763 | 243.000 | 0.067 | -3.349 | 1.975 | 186.000 | 0.092 |
|  | Bif | 1.365 | 1.878 | 243.000 | 0.468 | 2.393 | 2.187 | 186.000 | 0.275 |
